# STAT3 regulated ARF expression suppresses prostate cancer metastasis

**DOI:** 10.1038/ncomms8736

**Published:** 2015-07-22

**Authors:** Jan Pencik, Michaela Schlederer, Wolfgang Gruber, Christine Unger, Steven M. Walker, Athena Chalaris, Isabelle J. Marié, Melanie R. Hassler, Tahereh Javaheri, Osman Aksoy, Jaine K. Blayney, Nicole Prutsch, Anna Skucha, Merima Herac, Oliver H. Krämer, Peter Mazal, Florian Grebien, Gerda Egger, Valeria Poli, Wolfgang Mikulits, Robert Eferl, Harald Esterbauer, Richard Kennedy, Falko Fend, Marcus Scharpf, Martin Braun, Sven Perner, David E. Levy, Tim Malcolm, Suzanne D. Turner, Andrea Haitel, Martin Susani, Ali Moazzami, Stefan Rose-John, Fritz Aberger, Olaf Merkel, Richard Moriggl, Zoran Culig, Helmut Dolznig, Lukas Kenner

**Affiliations:** 1 Ludwig Boltzmann Institute for Cancer Research, Waehringerstrasse 13A, 1090 Vienna, Austria; 2 Clinical Institute of Pathology, Medical University of Vienna, 1090 Vienna, Austria; 3 Department of Molecular Biology, Paris-Lodron University of Salzburg, 5020 Salzburg, Austria; 4 Institute of Medical Genetics, Medical University of Vienna, 1090 Vienna, Austria; 5 Center for Cancer Research and Cell Biology, Queen's University Belfast, BT7 1NN Belfast, UK; 6 Institute of Biochemistry, University of Kiel, 24098 Kiel, Germany; 7 Department of Pathology and NYU Cancer Institute, NYU School of Medicine, New York 10016, USA; 8 Department of Microbiology and NYU Cancer Institute, NYU School of Medicine, New York 10016, USA; 9 NI Stratified Medicine Research Group, University of Ulster, BT47 6SB Londonderry, UK; 10 CeMM Research Center for Molecular Medicine of the Austrian Academy of Sciences, 1090 Vienna, Austria; 11 Department of Toxicology, University Medical Center, 55131 Mainz, Germany; 12 Molecular Biotechnology Center (MBC), Department of Genetics, Biology and Biochemistry, University of Turin, Turin 10126, Italy; 13 Department of Medicine I, Division: Institute for Cancer Research, Comprehensive Cancer Center, Medical University of Vienna, 1090 Vienna, Austria; 14 Department of Laboratory Medicine, Medical University of Vienna, 1090 Vienna, Austria; 15 Institute of Pathology and Neuropathology, University Hospital Tuebingen, 72076 Tuebingen, Germany; 16 Institute of Pathology, Center for Integrated Oncology Cologne/Bonn, University Hospital of Bonn, 53127 Bonn, Germany; 17 Department of Pathology, University of Cambridge, CB2 0QQ Cambridge, UK; 18 Department of Chemistry and Biotechnology, Swedish University of Agricultural Sciences, 75007 Uppsala, Sweden; 19 Unit for Translational Methods in Cancer Research, University of Veterinary Medicine Vienna, 1210 Vienna, Austria; 20 Department of Urology, Medical University of Innsbruck, 6020 Innsbruck, Austria; 21 Unit of Pathology of Laboratory Animals (UPLA), University of Veterinary Medicine Vienna, 1210 Vienna, Austria

## Abstract

Prostate cancer (PCa) is the most prevalent cancer in men. Hyperactive STAT3 is thought to be oncogenic in PCa. However, targeting of the IL-6/STAT3 axis in PCa patients has failed to provide therapeutic benefit. Here we show that genetic inactivation of *Stat3* or *IL-6* signalling in a *Pten*-deficient PCa mouse model accelerates cancer progression leading to metastasis. Mechanistically, we identify p19^ARF^ as a direct Stat3 target. Loss of Stat3 signalling disrupts the ARF–Mdm2–p53 tumour suppressor axis bypassing senescence. Strikingly, we also identify *STAT3* and *CDKN2A* mutations in primary human PCa. *STAT3* and *CDKN2A* deletions co-occurred with high frequency in PCa metastases. In accordance, loss of STAT3 and p14^ARF^ expression in patient tumours correlates with increased risk of disease recurrence and metastatic PCa. Thus, STAT3 and ARF may be prognostic markers to stratify high from low risk PCa patients. Our findings challenge the current discussion on therapeutic benefit or risk of IL-6/STAT3 inhibition.

## Abstract (web summary)

IL6-STAT3 signaling is activated in prostate cancer, however inhibiting this pathway has not lead to a survival advantage in patients. Here, Pencik *et al.* show that loss of the IL6-STAT3 axis in mice and humans leads to metastasis due to loss of ARF, unravelling STAT3 and ARF as potential prognostic markers in prostate cancer.

## Graphical abstract

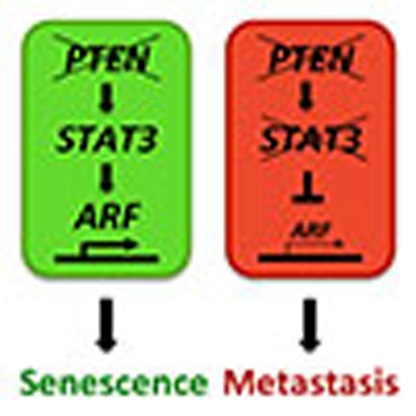

PCa is the second most frequently diagnosed cancer in men with >200,000 new cases reported in the USA annually^1^. Screening for the sensitive yet diagnostically unspecific biomarker prostate-specific antigen (PSA) has led to a substantial rise in the diagnosis of early stage PCa^2^. The failure of current diagnostic tools to reliably distinguish non-aggressive tumours from aggressive ones to predict therapeutic response^3^ urgently calls for the identification of better biomarkers to guide treatment. Furthermore, there is need for novel targeted therapies of metastatic PCa based on a better molecular understanding of the disease^4^. The lack of markers to stratify PCa cases into low- and high-risk groups results in overtreatment of 20–42% of patients^5^. STAT3, the major downstream mediator of IL-6 signalling, was shown to be related to advanced tumour growth, by tumour-autonomous mechanisms and by modulating tumour-associated stroma^6^. Although STAT3 activation is observed in ∼50% of PCa^7^ its functional role in tumorigenesis and metastasis has not been elucidated. Data from the majority of human PCa cancer cell lines support an oncogenic and growth promoting role of IL-6 and STAT3 *in vitro*^8^. However, metastatic LNCaP cells were growth inhibited *in vitro* and *in vivo* in response to IL-6 treatment^8^. Moreover, treatment of patients with an IL-6 blocking antibody did not result in a survival advantage in patients with advanced PCa^9^. Thus, addressing the precise *in vivo* role of IL-6/STAT3 in PCa is of utmost importance to reassess diagnostic and therapeutic approaches.

*PTEN* is one of the most frequently deleted or mutated tumour suppressors in PCa, with an estimated incidence of 70% in metastatic PCa, causing aberrant activation of the PI3K–AKT–mTOR signalling pathway^10^. Loss of *Pten* leads to senescence, which is critically regulated by the ARF–p53 pathway^11^. While the tumour suppressor ARF (p14^ARF^ in humans; p19^ARF^ in mice) is readily degraded in normal cells, it is stabilized to increase p53 function on loss of Pten. ARF was shown to augment p53 stability by promoting the degradation of Mdm2, a negative regulator of p53. Concomitant inactivation of *Pten* and *p53* leads to bypass of senescence and as a consequence to a malignant PCa phenotype^11^. Previous studies report PTEN–STAT3 signalling crosstalk in malignant glioblastoma^12^, but the detailed molecular mechanisms in cancer progression and metastasis remain unresolved.

In this study, we show that loss of IL-6/Stat3 signalling in a *Pten*-deficient PCa model accelerates cancer progression leading to metastasis. Loss of IL-6/Stat3 signalling in PCa bypasses senescence via disrupting the ARF–Mdm2–p53 tumour suppressor axis. We identify ARF as a novel direct Stat3 target. Notably, loss of STAT3 and p14^ARF^ expression correlates with increased risk of recurrence in PCa patients. In addition, STAT3 and p14^ARF^ expression was lost in metastasis compared with the primary tumours. We identified *STAT3* and *CDKN2A* mutations in primary PCa patients. Furthermore, PCa metastases show a high frequency of *STAT3* and *CDKN2A* deletions. We propose STAT3 and ARF as prognostic markers for high versus low risk PCa patient stratification.

## Results

### Co-deletion of *Stat3* and *Pten* triggers PCa

**Figure 1:**
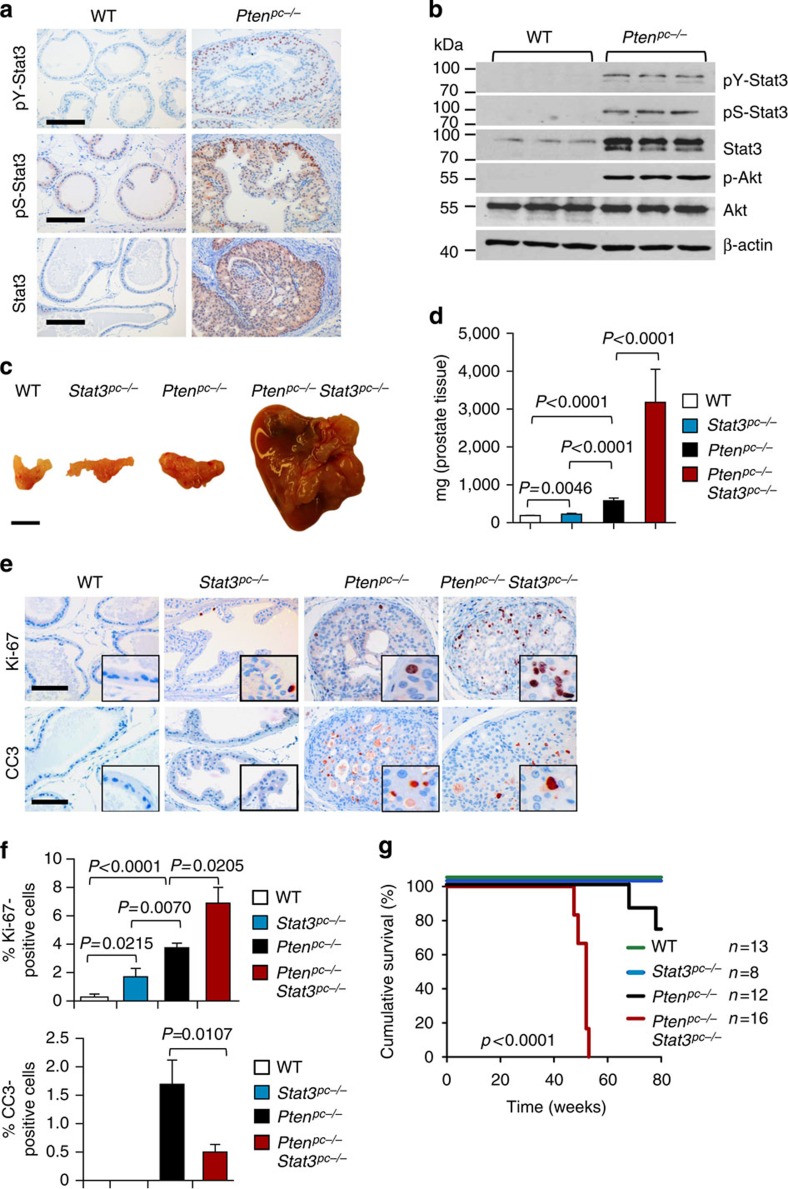
Genetic deletion of *Stat3* and *Pten* triggers progressive prostate tumorigenesis and lethal disease. (**a**) Comparison of prostates from WT and *Pten*^*pc−/−*^ mice at 19 weeks of age using immunohistochemical (IHC) analysis of pY-Stat3, pS-Stat3 and Stat3. Scale bars, 100 μm. (**b**) Protein expression analysis of pY-Stat3, pS-Stat3, Stat3, p-Akt, Akt and β-actin with western blots in 19-week-old prostates from WT and *Pten*^*pc−/−*^ mice. (**c**) Gross anatomy of representative prostates isolated at 52 weeks of age from WT, *Stat3*^*pc−/−*^, *Pten*^*pc−/−*^ and *Pten*^*pc−/−*^*Stat3*^*pc−/−*^ mice. Scale bars, 10 mm. (**d**) Prostate weights of 52-week-old WT, *Stat3*^*pc−/−*^, *Pten*^*pc−/−*^ and *Pten*^*pc−/−*^*Stat3*^*pc−/−*^ mice (*n*=24). Mean values are shown; Data were analysed by one-way analysis of variance with Tukey's multiple comparison test; error bars: s.d. (**e**) IHC analyses of prostates from 19-week-old WT, *Stat3*^*pc−/−*^, *Pten*^*pc−/−*^ and *Pten*^*pc−/−*^*Stat3*^*pc−/−*^ mice stained for Ki-67 and cleaved caspase 3 (CC3). Scale bars, 100 μm; insets: × 600 magnification. (**f**) Quantification of cells positive for Ki-67 and CC3 using HistoQuest software (*n*=5). Data were analysed by Student's *t*-test and are shown as mean±s.d. (**g**) Kaplan–Meier cumulative survival analysis revealed a significant (*P*<0.0001; log-rank test) decrease in lifespan of *Pten*^*pc−/−*^*Stat3*^*pc−/−*^ compared with *Pten*^*pc−/−*^ mice (*n*=49); WT and *Stat3*^*pc−/−*^ mice served as controls.

To study the role of PTEN and STAT3 in PCa development, we took advantage of mice with conditional loss of *Pten* in the prostate epithelium (*Pb-Cre4 Pten*^*fl/fl*^) hereafter referred to as *Pten*^*pc−/−*^ (ref. 13). Stat3 protein levels were markedly induced in *Pten*^*pc−/−*^ compared with wild-type (WT) prostate epithelium (Fig. 1a,b). *Pten*^*pc−/−*^ tumours showed strong Akt Ser473 phosphorylation and, unexpectedly, Stat3 Tyr705 and Ser727 phosphorylation suggesting maximal transcriptional activity of Stat3 (Fig. 1a,b and Supplementary Fig. 1a). In addition, we observed an increase in IL-6Rα levels in tumour cells and soluble IL-6R serum levels (Supplementary Fig. 1a–c) as well as increased *Stat3*, *IL-6Rα* and *IL-6* mRNA levels (Supplementary Fig. 1d) in *Pten*^*pc−/−*^ PCa compared with WT controls. To prove that loss of Stat3 signalling influences PCa formation, we generated mice with concomitant loss of *Pten* and *Stat3* in prostate epithelial cells. Prostate-specific deletion of *Pten* and *Stat3* was confirmed by PCR (Supplementary Fig. 2a). Immunohistochemistry (IHC) analysis confirmed loss of pY-Stat3 and Stat3 in *Pten*^*pc−/−*^*Stat3*^*pc−/−*^ tumour cells (Supplementary Fig. 2b), while still being present in stromal cells (Supplementary Fig. 2c). Surprisingly, and in sharp contrast to the oncogenic role of Stat3 in many cancers^14^,^15^, *Pten*^*pc−/−*^*Stat3*^*pc−/−*^ mice showed accelerated PCa formation with up to sixfold increase in tumour weight compared with *Pten*^*pc−/−*^ tumours at different stages of PCa development (Fig. 1c,d and Supplementary Fig. 2d,e). *Pten*^*pc−/−*^ *Stat3*^*pc−/−*^ tumours showed increased numbers of Ki-67 positive (Ki-67^+^) proliferating cells and reduced numbers of cleaved caspase 3 positive (CC3^+^) apoptotic cells compared with *Pten*^*pc−/−*^ prostates (Fig. 1e,f).

**Figure 2:**
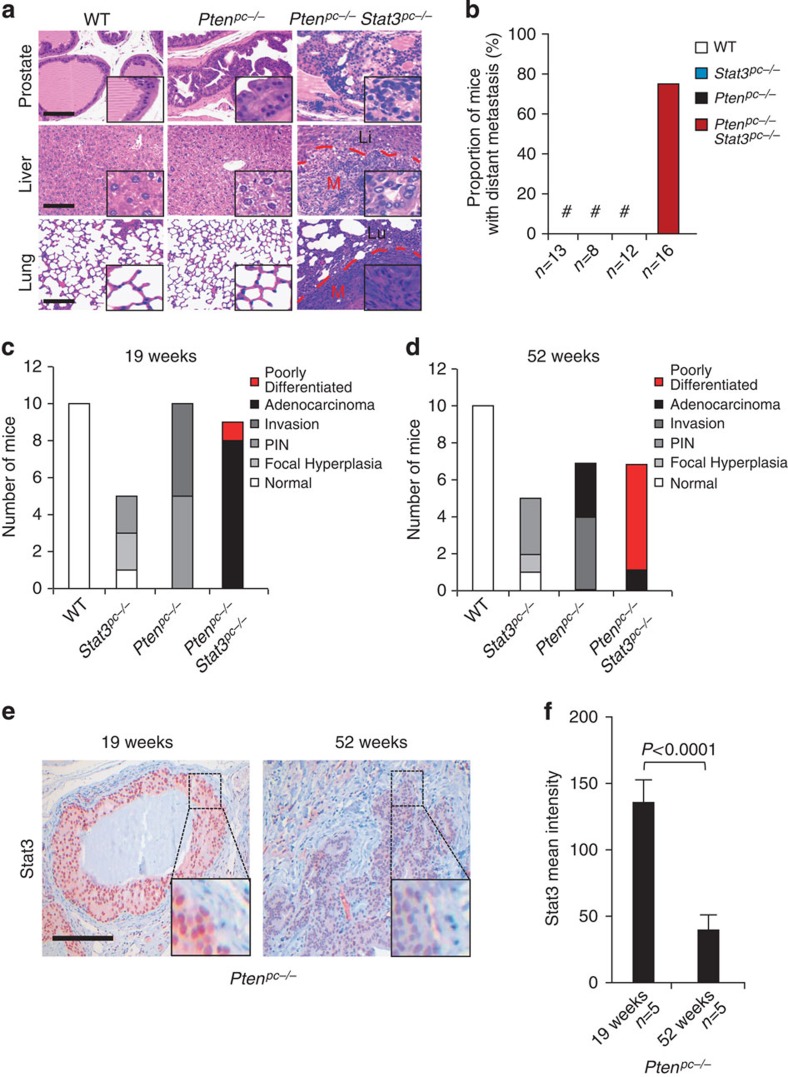
Co-deletion of *Stat3* and *Pten* enhances prostate cancer transformation and metastatic potential. (**a**) Histopathological analysis of primary PCa, livers and lungs at 52 weeks of age from WT, *Pten*^*pc−/−*^ and *Pten*^*pc−/−*^*Stat3*^*pc−/−*^ mice. The dashed red lines encircle areas of advanced liver or lung metastases (M), which are surrounded by normal liver (Li) or normal lung (Lu), respectively. Scale bars, 100 μm; insets: × 600 magnification. (**b**) Percentage of mice with sites of distant PCa metastases (*n*=49). (**c**) Summary of the histological findings of mouse prostates examined at 19 weeks postpartum (p.p.) from WT, *Stat3*^*pc−/−*^*, Pten*^*pc−/−*^ and *Pten*^*pc−/−*^*Stat3*^*pc−/−*^ mice. (**d**) Summary of the histological findings of mouse prostates examined at 52 weeks p.p. from WT, *Stat3*^*pc−/−*^*, Pten*^*pc−/−*^ and *Pten*^*pc−/−*^*Stat3*^*pc−/−*^ mice. Histological grading and classification of mouse prostates was done according to Chen *et al.*^67^ (**e**) Stat3 IHC in 19-week- and 52-week-old *Pten*^*pc−/−*^ prostate tumours. Scale bars, 100 μm. (**f**) Quantification of Stat3 staining in 19-week- and 52-week-old *Pten*^*pc−/−*^ prostate tumours using HistoQuest software, *P*<0.0001. Data were analysed by Student's *t*-test and are shown as mean±s.d. (*n*=5).

Compared with *Pten*^*pc−/−*^ mice^16^, *Pten*^*pc−/−*^*Stat3*^*pc−/−*^ mice displayed a significantly reduced median survival (Fig. 1g). Intriguingly, *Pten*^*pc−/−*^*Stat3*^*pc−/−*^ mice developed high-grade (poorly differentiated) PCa with liver and lung metastases (Fig. 2a). Histopathological analysis of PCa-bearing animals revealed widespread metastasis in 75% of *Pten*^*pc−/−*^*Stat3*^*pc−/−*^ mice. By contrast, *Pten*^*pc−/−*^ mice only showed local invasion into seminal vesicles and never developed distant metastases (Fig. 2b and Supplementary Table 1). Furthermore, loss of Stat3 promoted PCa formation in Pten heterozygous prostate tissue (*Pten*^*pc+/−*^*Stat3*^*pc−/−*^) at 19 weeks of age, whereas *Pten*^*pc+/−*^mice developed only prostatic intraepithelial neoplasia^17^ (PIN) (Supplementary Fig. 3a). Our data demonstrate that Stat3 suppresses malignant progression of *Pten*-deficient PCa. We found reduced p53 protein expression in *Pten*^*pc+/−*^*Stat3*^*pc−/−*^ prostates (Supplementary Fig. 3b). Pten heterozygosity alone had no effect on p53 expression as demonstrated before^11^. Interestingly, prostate-specific loss of *Stat3* (*Stat3*^*pc−/−*^) led to development of PIN lesions in prostates at 19 weeks of age (Supplementary Fig. 3c). Analysis of consecutive sections revealed invasive regions and high-grade PIN in *Pten*^*pc−/−*^ mice at 19 weeks of age, whereas *Pten*^*pc−/−*^*Stat3*^*pc−/−*^ mice showed early progression to poorly differentiated adenocarcinoma (Fig. 2c). At 52 weeks of age *Pten*^*pc−/−*^*Stat3*^*pc−/−*^ tumours had developed into poorly differentiated carcinomas with 86% penetrance compared with *Pten*^*pc−/−*^ tumours, which showed only signs of focal invasion and late adenocarcinoma formation (Fig. 2d). Notably, *Pten*^*pc−/−*^ tumour cells uniformly expressed nuclear Stat3 at 19 weeks of age (Fig. 2e), whereas more advanced tumours at 52 weeks of age showed profoundly reduced nuclear Stat3 expression (Fig. 2e,f), suggesting that Stat3 expression decreased during PCa progression.

**Figure 3:**
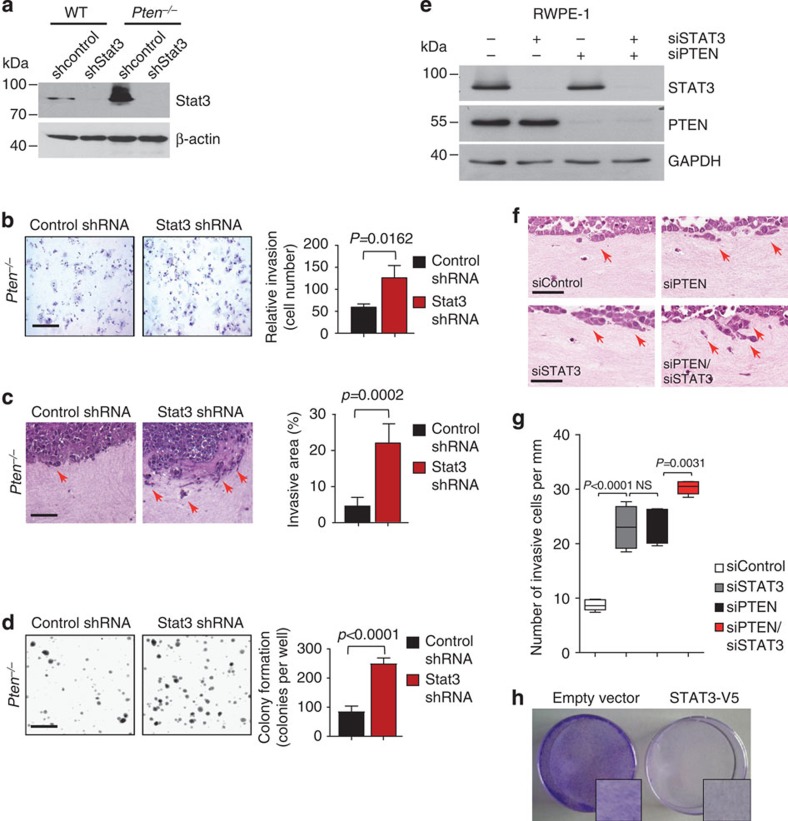
*Stat3* suppresses colony formation and invasion. (**a**) shRNA-mediated knockdown of Stat3 in *Pten*^*−/−*^ mouse PCa cells leads to robust decrease of Stat3 levels as demonstrated by western blotting. In all knockdown experiments, scrambled non-target shRNA served as a control (control shRNA/shcontrol). (**b**) Representative histology of increased matrigel invasion after shRNA-mediated knockdown of Stat3 in *Pten*-deficient (*Pten*^*−/−*^) mouse PCa cells. Quantification of relative invasion is shown (*n*=3). Scale bars, 100 μm. (**c**) Organotypic culture assays of *Pten*^*−/−*^ mouse PCa cells in the absence of Stat3 showed capacity to invade into the fibroblast containing collagen gel (red arrows). Scale bars, 50 μm. The invasive area/total tumour cell area was quantified (control shRNA, *n*=4, shStat3 *n*=3). (**d**) Soft agar colony formation of primary *Pten*^*−/−*^ mouse PCa cells with shStat3 and controls was quantified (*n*=3). Scale bars, 200 μm. Data were analysed by Student's *t*-test and are shown in **c**–**e** as mean±s.d. (**e**) Efficient STAT3 and PTEN siRNA-mediated knockdown of RWPE-1 cells was demonstrated by western blot. Scrambled non-target siRNA served as a control (control siRNA/siControl) (**f**) Organotypic culture of RWPE-1 cells in the presence and absence of STAT3 and/or PTEN cultivated in contact with human prostate stromal fibroblasts seeded in collagen I gels after 8 days of culture. Representative H&E stainings are shown, red arrows indicate invasion, scale bars, 100 μm. (*n*=3). (**g**) Quantification of invasion of RWPE-1 cells knocked down for PTEN and/or STAT3 using siRNA (number of invasive cells per mm, *n*=5 sections for each condition). Data were analysed by one-way analysis of variance with Tukey's multiple comparison test; error bars: s.d. (**h**) Crystal violet stains of focus formation of STAT3-V5 and empty vector transduced PC3 cells after 4 days of incubation (Supplementary Fig. 4c–e).

To further dissect the tumour promoting effects of loss of Stat3, we established primary mouse *Pten*^*−/−*^ PCa cells with stable, short hairpin RNA (shRNA) mediated knockdown of *Stat3*. Western blot and IHC analyses confirmed efficient Stat3 knockdown in these cells (Fig. 3a and Supplementary Fig. 4a). In line with our genetic data, *Pten*^*−/−*^ mouse PCa cells with shStat3 knockdown were significantly more invasive in a transwell invasion assay compared with control cells (Fig. 3b). We verified the aggressive behaviour of *PTEN–STAT3* double deficient tumour cells in an organotypic, physiologically relevant *in vitro* three-dimensional cancer model^18^. *Pten*^*−/−*^*-*shStat3 PCa cells displayed a more invasive phenotype compared with control cells in organotypic assays (Fig. 3c and Supplementary Fig. 4b). In addition, *Pten*^*−/−*^-shStat3 cells showed increased anchorage-independent cell growth in soft agar compared with *Pten*^*−/−*^ PCa cells expressing non-targeting shRNA (control shRNA) (Fig. 3d). To corroborate our findings in human cells, combined knockdown of STAT3 and PTEN in human RWPE-1 prostate cells (Fig. 3e) increased invasiveness in organotypic assay compared with control and single knockdown cells (Fig. 3f,g). Notably, re-expression of STAT3 in human PC3 prostate carcinoma cells, which lack STAT3 expression (Supplementary Fig. 4c), led to significantly decreased cell numbers and reduced foci formation (Fig. 3h and Supplementary Fig. 4d,e). These data are consistent with a cell-autonomous tumour suppressive role of Stat3 in PCa.

### Stat3 regulates the ARF–Mdm2–p53pathway

**Figure 4:**
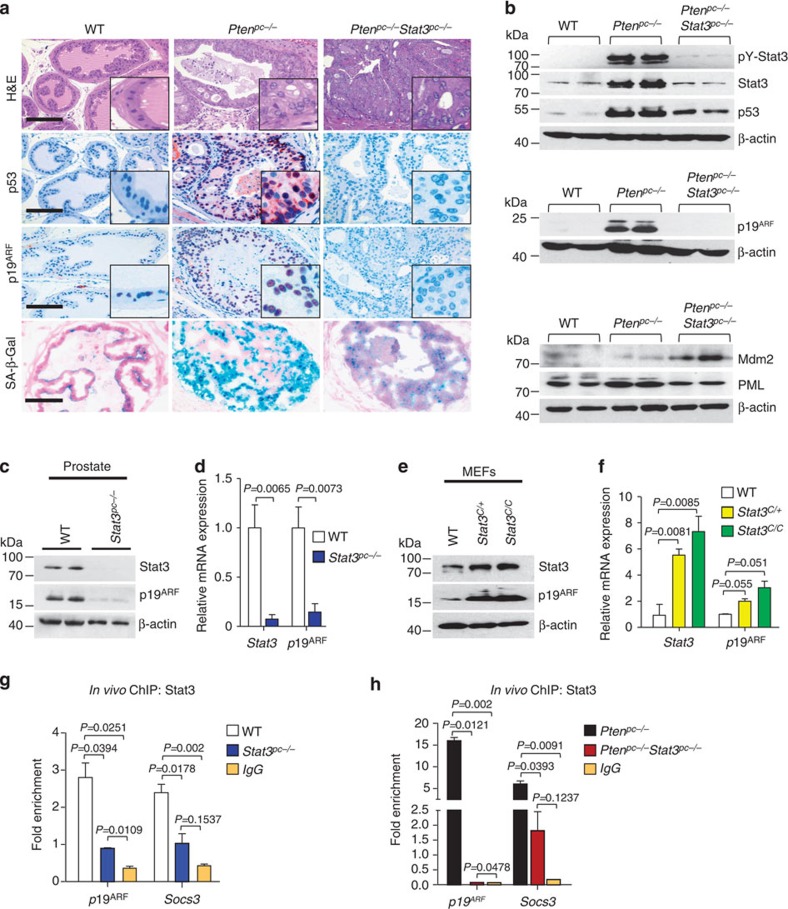
Stat3 is a critical regulator of the ARF–Mdm2–p53 tumour suppressor pathway and senescence. (**a**) Haematoxilin/eosin (H&E) stains show higher grade PCa in *Pten*^*pc−/−*^*Stat3*^*pc−/−*^ mice compared with *Pten*^*pc−/−*^ mice. Scale bars, 100 μm. IHC analysis of p53, p19^ARF^ and staining for senescence-associated-β-galactosidase activity in prostates from 19-week-old WT, *Pten*^*pc−/−*^ and *Pten*^*pc−/−*^ *Stat3*^*pc−/−*^ mice. Scale bars, 100 μm; insets: × 600 magnification. (**b**) Western blot analysis showing pY-Stat3, Stat3, p53, p19^ARF^, Mdm2 and PML expression levels in *Pten*^*pc−/−*^*Stat3*^*pc−/−*^ compared with *Pten*^*pc−/−*^ mice. β-actin serves as a loading control. The remaining Stat3 bands in *Pten*^*pc−/−*^*Stat3*^*pc−/−*^ prostates are due to Stat3 stromal expression (Supplementary Fig. 2c). (**c**) Western blot analysis of Stat3 and p19^ARF^ expression in prostates of 19-week-old WT or *Stat3*^*pc−/−*^ mice. β-actin serves as a loading control. (**d**) qRT–PCR analysis of *Stat3* and *p19*^*ARF*^ mRNA expression in prostates of 19-week-old WT or *Stat3*^*pc−/−*^ mice (*n*=5 each). Data were analysed by Student's *t*-test and are shown as mean±s.d. (**e**) Western blot analysis of Stat3 and p19^ARF^ expression in WT, *Stat3*^*C/+*^ or *Stat3*^*C/C*^ MEFs. (**f**) qRT–PCR analysis of *Stat3* and *p19*^*ARF*^ transcript levels in WT, *Stat3*^*C/+*^ and *Stat3*^*C/C*^ MEFs (*n*=3 each). Data were analysed by Student's *t*-test and are shown as mean±s.d. (**g**) *In vivo* ChIP analysis of Stat3 binding to *p19*^*ARF*^ and *Socs3* promoters, respectively, in WT and *Stat3*^*pc−/−*^ prostate tissue. Stat3 binding to the *Socs3* (ref. 69) promoter, which is a direct Stat3 target served as a positive control. Data were normalized to Cis4200, which served as the negative control^70^. (**h**) *In vivo* ChIP analysis of Stat3 binding to the *p19*^*ARF*^ and *Socs3* promoters in PCa. Note the >15-fold enrichment of Stat3 bound to promoter fragments in *Pten*^*pc−/−*^ compared with *Pten*^*pc−/−*^*Stat3*^*pc−/−*^ tumours. Data in **g** and **h** were analysed by one-way analysis of variance with Tukey's multiple comparison test and shown as mean±s.d. (Primer pairs are listed in Supplementary Table 2).

Since loss of *Pten* triggers senescence thereby restricting cancer progression and metastasis^11^, we next tested whether Stat3 exerts a tumour suppressive function by activating senescence-inducing programmes in *Pten*^*pc−/−*^PCa cells^19^ at an early stage of PCa development (19 weeks). Senescence is generally characterized by upregulation of p53, cyclin-dependent kinase inhibitor 1 (Cdkn1, p21), promyelocytic leukaemia protein (PML) and elevated senescence-associated-β-galactosidase activity^20^. Of note, *Pten*^*pc−/−*^*Stat3*^*−/−*^ tumours lacked p21 expression, displayed reduced numbers of PML nuclear bodies and decreased SA-β-Gal activity compared with *Pten*^*pc−/−*^ tumours (Fig. 4a,b and Supplementary Fig. 5a,b), suggesting Stat3 as a novel mediator of senescence in response to loss of Pten. Senescence associated with loss of Pten was shown to be bypassed by deletion of p53 leading to early lethality^11^. We show here that loss of Stat3 and Pten revealed a phenotype strikingly similar to that of p53 and Pten loss^11^. Intriguingly, Stat3 and Pten deletion resulted in downregulation of p53 expression in the prostate epithelium, which was accompanied by the loss of p19^ARF^ (Fig. 4a,b). The p53 expression in the tumour stromal cells remained unchanged (Supplementary Fig. 5c). Since p19^ARF^ is a critical regulator of Mdm2 degradation^21^, our results suggest that the tumour suppressive capacity of Stat3 in senescent tumour cells^22^ may rely on the p19^ARF^–Mdm2–p53 tumour suppressor axis.

Recently, co-inactivation of p53 and ARF was linked to human triple-negative breast cancer progression through IFNβ–STAT1–ISG15 signalling^23^. Interestingly, ISG15 and Stat3 protein expression levels paralleled each other in the PCa mouse models (Supplementary Fig. 6a). This suggests an altered ISGylation system, which could be independent of androgen receptor expression^24^ (Supplementary Fig. 6b). It is noteworthy, that numerous AR-positive tumour cells were observed in the metastases of the *Pten*^*pc−/−*^*Stat3*^*pc−/−*^ PCa mouse models (Supplementary Fig. 6c), underlining the relevance of our model to human PCa^25^. Molecular pathological analysis confirmed AR-positive tumour nodules in five out of five cases of lung metastases consist of 66% AR-positive cells (Supplementary Fig. 6d).

Mechanistically, Pten was shown to block PI3K-mediated translocation of Mdm2 into the nucleus, where it promotes rapid degradation of p53 (ref. 26). In line with the published function of PML to increase p53 stability by sequestering Mdm2 to the nucleolus, disruption of *Pten* and *Stat3* resulted in increased Mdm2 protein levels (Fig. 4b). Therefore, loss of *Stat3* promotes PCa development by bypassing senescence regulated by the p19^ARF^–p53 axis. We therefore hypothesize that Stat3 may act through ARF as an important gatekeeper controlling senescence counteracting metastasis, which we tested further.

Immunoblot and IHC analyses revealed a positive correlation between Stat3 and p19^ARF^ levels. Moreover, loss of Stat3 led to a profound decrease in p19^ARF^ protein (Fig. 4a,b). Transcript levels of the well-established Stat3 target genes *Socs3* and *c-fos*^27^ paralleled p19^ARF^ expression in WT, *Pten*^*pc−/−*^ and *Pten*^*pc−/−*^*Stat3*^*pc−/−*^ prostate tissue (Supplementary Fig. 7a,b). *Stat3*^*pc−/−*^ prostates displayed a significant decrease in p19^ARF^ protein and mRNA levels compared with WT controls (Fig. 4c,d), suggesting direct regulation of p19^ARF^ expression by Stat3. We first tested this by analysing p19^ARF^ expression in passage-matched homozygous *Stat3* knockout (*Stat3*^*KO*^) and WT mouse embryonic fibroblasts (MEFs). Both p19^ARF^ protein and mRNA levels were significantly reduced in the absence of Stat3 (Supplementary Fig. 7c,d). Consistently, MEFs expressing one or two hyperactive Stat3 alleles (*Stat3*^*C/+*^ and *Stat3*^*C/C*^) from the endogenous Stat3 locus^28^ displayed upregulation of p19^ARF^ protein and mRNA (Fig. 4e,f). *In silico* analysis of the *p19*^*ARF*^ promoter^29^ predicted two potential Stat3-binding sites (Supplementary Fig. 7e). Chromatin immunoprecipitation (ChIP) experiments showed approximately threefold increase in Stat3-binding activity to the proximal p19^*ARF*^ promoter region in WT compared with *Stat3*^*pc−/−*^ prostate tissue (Fig. 4g). We next wanted to know if Stat3 binding was further elevated in *Pten*-deficient tumours since *Pten* loss increased ARF expression. By performing ChIP in *Pten*^*pc−/−*^ primary mouse prostate tumours, we demonstrated a 15-fold increase of Stat3 binding to the *p19*^*ARF*^ promoter (Fig. 4h), which was abrogated in *Pten*^*pc−/−*^*Stat3*^*pc−/−*^ tumours. These data identify p19^ARF^ as a novel Stat3 target gene.

### Loss of *IL-6* and *Pten* leads to cancer and metastasis

**Figure 5:**
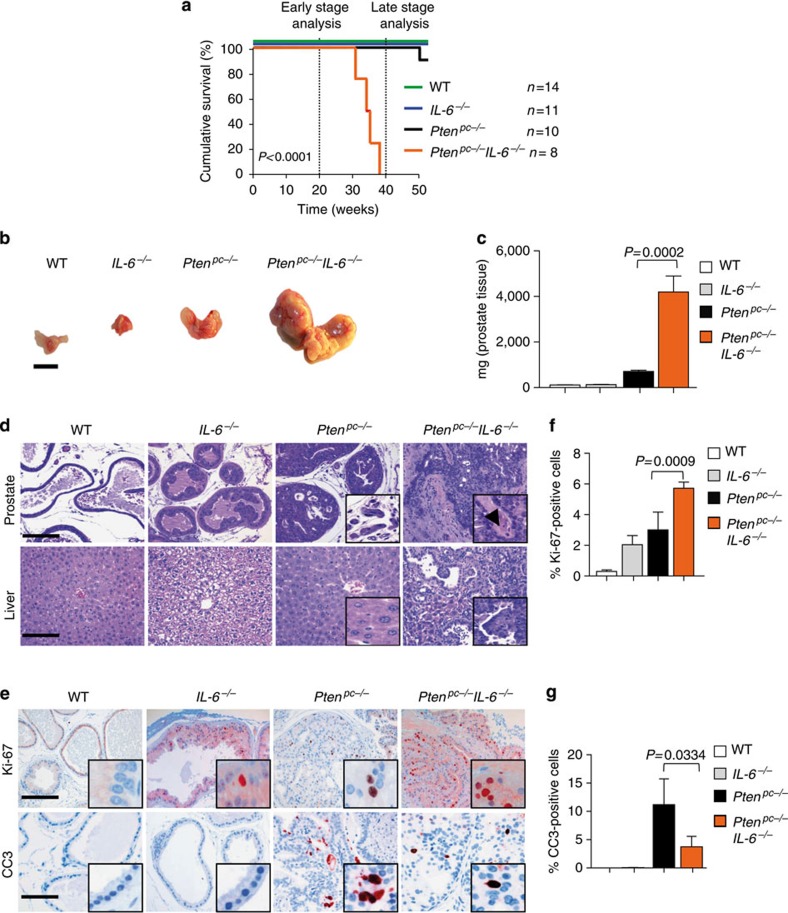
Deletion of *IL-6* and *Pten* triggers progressive prostate tumorigenesis and metastatic disease. (**a**) Kaplan–Meier cumulative survival analysis of *Pten*^*pc−/−*^*IL-6*^*−/−*^ compared with *Pten*^*pc−/−*^ mice; WT and *IL-6*^*−/−*^ mice served as controls (*P*<0.0001; log-rank test). (**b**) Gross anatomy of representative prostates isolated at 38 weeks of age from WT, *IL-6*^*−/−*^, *Pten*^*pc−/−*^ and *Pten*^*pc−/−*^*IL-6*^*−/−*^ mice. Scale bars, 10 mm. (**c**) Prostate weights of 38-week-old WT, *IL-6*^*−/−*^*, Pten*^*pc−/−*^ and *Pten*^*pc−/−*^*IL-6*^*−/−*^ mice. Mean values are shown; error bars: s.d. (*n*=43). (**d**) Histopathological analysis of haematoxilin/eosin-stained primary PCa and liver at 38 weeks of age from WT, *IL-6*^*−/−*^, *Pten*^*pc−/−*^ and *Pten*^*pc−/−*^*IL-6*^*−/−*^ mice. Arrowhead in the inset: area of nerve sheet infiltration. Scale bars, 100 μm. (**e**) IHC analysis of Ki-67 and CC3 in prostates from 19-week-old WT, *IL-6*^*−/−*^, *Pten*^*pc−/−*^ and *Pten*^*pc−/−*^*IL-6*^*−/−*^ mice. Scale bars, 100 μm. (**f**,**g**) Bar graphs indicate percentage of cells positive for Ki-67 and CC3 (e). Protein levels quantification was done with HistoQuest software (*n*=5). Data from **c**, **f** and **g** were analysed by Student's *t*-test and are shown as mean±s.d.

IL-6 signalling is the major regulator of Stat3 with therapeutic relevance. To address whether Stat3 activation in *Pten*^*pc−/−*^ mice depends on IL-6 signalling, we crossed *Pten*^*pc−/−*^ mice with *IL-6*^*−/−*^ mice^30^. Co-deletion of *IL-6* and *Pten* triggered early lethality (Fig. 5a), progressive high-grade adenocarcinoma formation with increased tumour growth and weight (Fig. 5b,c), resulting in disseminated metastases (Fig. 5d), for example, in the liver (Supplementary Fig. 8a). Loss of *IL-6* in *Pten* heterozygous prostate tissue also resulted in high-grade invasive PCa with metastasis formation at 15 months of age (Supplementary Fig. 8b). Like *Pten*^*pc−/−*^*Stat3*^*pc−/−*^ mice, *Pten*^*pc−/−*^*IL-6*^*−/−*^ mice showed markedly enhanced PCa growth (Supplementary Fig. 8c,d) at 19 weeks of age, increased Ki-67^+^ but decreased CC3^+^ expression levels compared with *Pten*^*pc−/−*^ prostates (Fig. 5e–g). IHC analysis revealed absence of pY-Stat3 and Stat3 expression in *Pten*^*pc−/−*^*IL-6*^*−/−*^ tumours (Supplementary Fig. 8e).

**Figure 6:**
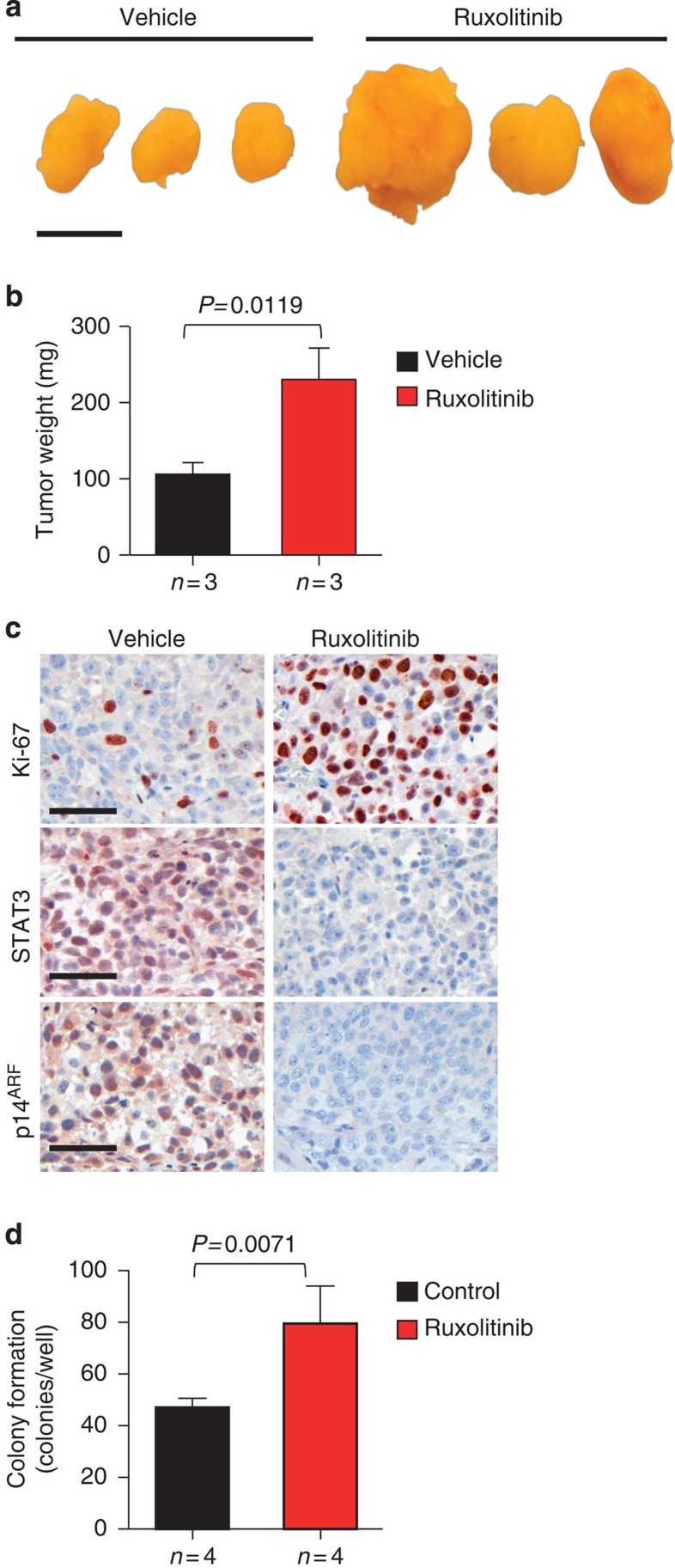
JAK1/2 inhibition promotes tumour progression and decreases STAT3 and p14^ARF^ expression. (**a**) Gross anatomy of representative LNCaP xenograft tumours treated with ruxolitinib. Mice bearing xenografts were treated with a vehicle or 50 mg kg^−1^ ruxolitinib. Scale bars, 10 mm. (**b**) Tumour weight of vehicle-treated mice versus ruxolitinib treatment for 22 days of age-matched SCID beige mice. Mean values are shown; error bars: s.d. (*n*=3). (**c**) IHC stainings of Ki-67, STAT3 and p14^ARF^ expression in vehicle versus ruxolitinib-treated xenografted tumours (*n*=3), scale bar 50 μm. (**d**) LNCaP cells treated with control (DMSO) or 10 μM ruxolitinib were grown in soft agar for 12 days. Mean values are shown; error bars: s.d. (*n*=4). Data from **b** and **d** were analysed by Student's *t*-test.

Intriguingly, *in vivo* blocking of IL-6/STAT3 signalling by the JAK1/2 inhibitor ruxolitinib in human LNCaP xenografts showed significantly enhanced tumour size and weight (Fig. 6a,b). The ruxolitinib-treated xenografts also had markedly increased numbers of Ki-67^+^ cancer cells accompanied by a decrease in STAT3 and p14^ARF^ expression (Fig. 6c). Moreover, ruxolitinib treatment of LNCaP cells significantly promoted colony formation (Fig. 6d). Of note, LNCaP cells lack JAK1 expression and do not respond to interferon signalling^31^. Moreover, STAT3 was shown to be activated in xenografts of LNCaP cells, most likely due to the sole action of JAK2 (refs 32, 33). Ruxolitinib is a dual-specific JAK1/2 kinase inhibitor and therefore, effects of ruxolitinib on the LNCaP xenograft model are likely to be a consequence of JAK2/STAT3 inhibition independent of IFN gamma signalling.

### Loss of STAT3 and ARF in PCa is associated with metastases

**Figure 7:**
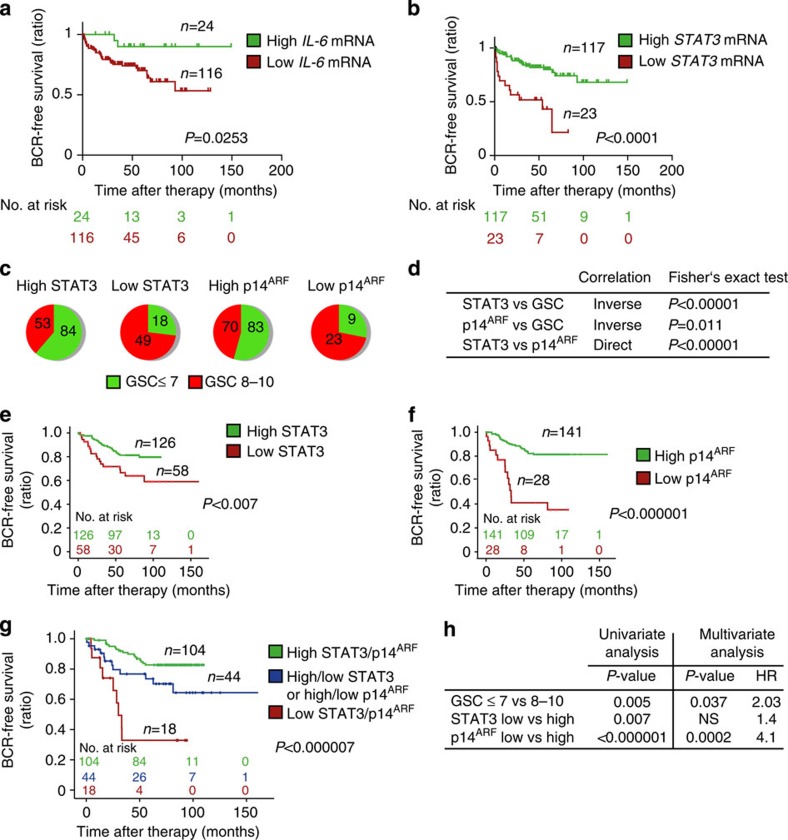
Loss of STAT3 and/or p14^ARF^ expression predicts early BCR in patients with PCa. (**a**,**b**) Kaplan–Meier analysis including number at risk of patients stratified into high or low *lL6-* and *STAT3* mRNA expression predicting biochemical recurrence (BCR) of the Taylor data set^35^. (**c**) Distribution of STAT3 and p14^ARF^ protein expression with low (≤7) or high (8–10) GSC in tumour specimens from men diagnosed with PCa. (**d**) Fisher's exact test of data shown in **c** and correlation of STAT3 to p14^ARF^ expression. (**e**) Kaplan–Meier analysis of BCR-free survival ratio based on STAT3 protein expression in a panel of 204 PCa patients. (**f**) Kaplan–Meier analysis of BCR-free survival ratio based on p14^ARF^ protein expression in a panel of 204 PCa patients. (**g**) Co-expression analyses of BCR-free survival of STAT3 and p14^ARF^. (**h**) Univariate and multivariate analyses of GSC, STAT3 or p14^ARF^ protein levels. Data from **a**,**b**,**e**,**f** and **g** were analysed by log-rank test.

The fact that activated STAT3 induces its own transcription^34^ led us to measure *IL-6* and *STAT3* mRNA levels in primary human tumours and to correlate them with clinical outcome. Using the Taylor gene expression profiling data set (GSE21032)^35^, we dichotomized samples based on the *z*-scores *of IL-6* or *STAT3* expression. Specifically for *IL-6* expression, samples that had *z*-scores<−2 were defined as low *IL-6* and all others (*z*-scores>−2) were used as a comparator for prognostic significance. Conversely, samples that had *STAT3 z*-scores>2 were defined as *STAT3* high and all other samples (*z*-scores<2) were used as comparators. Time to biochemical recurrence (BCR) was assessed as an indicator of individual prognosis. BCR was defined by an increase to >0.2 ng ml^−1^ PSA in serum. Of note, patients with low *IL-6* expression levels showed a significantly higher risk of BCR compared with patients with high *IL-6* expression (Fig. 7a). In line, patients with high *Stat3* mRNA levels demonstrate good prognosis. This may reflect a higher level of senescence competence compared with patients with lower *Stat3* mRNA levels (Fig. 7b).

**Figure 8:**
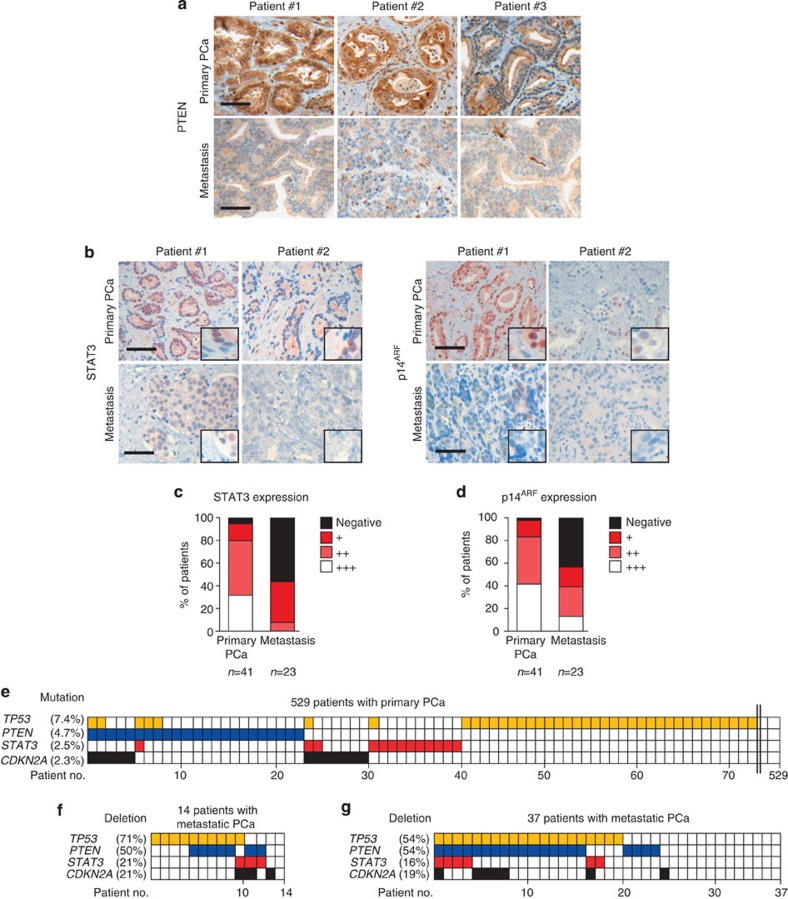
Loss of STAT3 and ARF in metastases of PCa patients. (**a**) Three representative images of PTEN expression determined by IHC analyses in matched patient samples with primary and metastatic PCa (*n*=5). Scale bars, 100 μm. (**b**) Representative IHC images of STAT3 and p14^ARF^ expression from primary (*n*=41) and metastatic (*n*=23) PCa samples. Scale bars, 100 μm. (**c**,**d**) STAT3 and p14^ARF^ staining intensity ranging from undetectable (Negative) to maximal expression levels (+++) in cohorts of primary and metastatic PCa. (**e**) Graphical representation of *TP53*, *PTEN*, *STAT3* and *CDKN2A* mutations in 529 patients with primary PCa^71^. (**f**) Graphical representation of *TP53*, *PTEN*, *STAT3*, *CDKN2A* deletions in 14 metastatic PCa^55^. Data were processed by Affymetrix Genome-Wide Human SNP Array 6.0. (**g**) Graphic representation of gene deletion analysis of *TP53*, *PTEN*, *STAT3* and *CDKN2A* in an independent data set of 37 metastatic PCa samples^35^.

We next tested whether expression of STAT3 and p14^ARF^ can serve as novel prognostic markers predicting the risk of BCR and metastatic disease progression. STAT3 and p14^ARF^ levels were independently evaluated by five pathologists in 204 human PCa specimens from patients who underwent radical prostatectomy. Patient samples were categorized into low or high expression groups based on staining intensities and number of positive cells (Methods) using validated antibodies (Supplementary Fig. 9a,b). Classification was verified by quantitative histopathological image analysis^36^ (Supplementary Fig. 10a). Indeed, increased Gleason score (GSC) correlated with low STAT3 or p14^ARF^ protein expression levels. In addition, we found a strong direct correlation between STAT3 and p14^ARF^ in accordance with p14^ARF^ being a direct target of STAT3 (Fig. 7c,d). STAT3 expression was significantly decreased in PCa (GCS≤7) compared with PIN areas of 67 matched patient samples (Supplementary Fig. 10b,c). Strikingly, both low STAT3 expression (*P*<0.007; log-rank test) and low p14^ARF^ (*P*<0.000001; log-rank test) expression were associated with poor outcome (Fig. 7e,f). Moreover, combined loss of p14^ARF^ and STAT3 expression in tumours of PCa patients predicted the worst outcome (*P*=0.000007, log-rank test) (Fig. 7g). Using multivariate analysis, we identified p14^ARF^ as a reliable, independent prognostic marker for PCa, which has a twofold higher hazard ratio compared with GSC (Fig. 7h). In accordance with the literature^25^,^37^, we found increased AR expression in metastases compared with primary human PCa (Supplementary Fig. 11a,b). IHC analysis of matched primary and metastatic human PCa revealed significant loss of PTEN expression in metastatic PCa (Fig. 8a and Supplementary Fig. 11c), suggesting an important role of PTEN loss in PCa metastasis. PTEN levels showed no significant correlation with STAT3 or p14^ARF^ protein expression in a cohort of >200 patients (Supplementary Fig. 11d,e). Moreover, we demonstrated that loss of STAT3 and/or p14^ARF^ is associated with progression to metastatic disease (Fig. 8b–d). This is supported by expression data from matched primary and metastatic PCa samples (Supplementary Fig. 12a–c), where in all seven cases a significant loss of STAT3 and p14^ARF^ expression occurred in the metastases.

Current data suggest that PCa harbour common genomic and epigenomic alterations^38^,^39^. We analysed large-scale human cancer data sets (International Cancer Genome Consortium (ICGC) and COSMIC databases) and the GEO methylation database^40^. Surprisingly, we found mutations of *STAT3* (2.5%) and *CDKN2A* (2.3%) in a cohort of 529 patients with primary PCa (Fig. 8e). We found five missense-, one nonsense- and several conservative *STAT3* mutations in primary PCa patients (Supplementary Fig. 13a). *STAT3* and/or *CDKN2A* were frequently deleted in metastatic PCa (Fig. 8f and Supplementary Fig. 13b), which have been confirmed in an independent data set^35^ (Fig. 8g). Notably, in 66% of STAT3 deletions, PTEN was co-deleted in both metastases data sets investigated (Fig. 8f,g). However, we neither detected human *IL-6* mutations nor methylation of *STAT3* and *CDKN2A* loci in primary and metastatic PCa (Supplementary Figs 13c,d and Supplementary Figs 14a,b).

## Discussion

Due to the lack of prognostic markers for risk stratification, many PCa patients suffer from overtreatment and a severely reduced quality-of-life. Understanding the molecular genetics underlying PCa and the identification of genetic and/or biochemical markers predicting clinical outcome is therefore of high priority.

Our data reveal robust upregulation of the IL-6/Stat3 signalling axis in a PCa mouse model as well as in patient specimens. Loss of *Stat3* or *IL-6* accelerates the progression to metastatic PCa; this stands in sharp contrast to the proposed oncogenic function of IL-6/Stat3 signalling in PCa^33^,^41^,^42^. Moreover, there are contradictory studies on the role of senescence in tumorigenesis^42^,^43^,^44^. Whether these different findings are due to the use of distinct mouse models, strains and species remains to be addressed. Here we combine an array of experimental approaches including *in vivo* models, mechanistic data with comprehensive human pathological, molecular and genetic data to underscore the tumour suppressive role of STAT3 (ref. 45). Thereby we identify STAT3 and its transcriptional target ARF as novel powerful prognostic markers for PCa patients. In PCa and several other cancers, elevated serum IL-6 levels correlate with a poor prognostic outcome for patients^46^. This sparked the development of IL-6 inhibitors to abrogate hyperactive IL-6/STAT3 signalling. However, blocking IL-6/STAT3 signalling was ineffective in patients with advanced PCa^9^,^47^, challenging this oncogenic concept.

We show that loss of Stat3 accelerates malignant progression of prostate tumours through abrogation of *p19*^*ARF*^ expression, which we identified as a novel direct Stat3 target gene. Several reports have demonstrated that loss of *Pten* leads to increased senescence associated with p53 stabilization triggered by p19^ARF^ (refs 11, 48). Inactivation of *Stat3* and *Pten* revealed striking similarities to the malignant phenotype observed after deletion of *Pten* and *p53* (ref. 11). Our model implicates high Stat3 and/or IL-6 levels in senescence-competent PCa and therefore does not contradict the general observation of high IL-6 and/or Stat3 expression in many cancers. We show that impaired *p19*^*ARF*^ expression in *Stat3-*deficient PCa is accompanied by loss of senescence. In line with our data, concomitant loss of *Pten* and *p19*^*ARF*^ in MEFs resulted in bypass of senescence, induction of hyperproliferation and oncogenic transformation^11^. Accordingly, overexpression of p19^ARF^ leads to p53-dependent cell growth arrest and induces senescence^49^. However, systemic *p19*^*ARF*^ deficiency in *Pten*^*pc−/−*^ mice did not accelerate PCa or enhance p53 accumulation^50^, which might be explained by inflammatory cytokines and/or growth factors produced by the multiple tumours developing in other organs^51^. Here we identified an unpredicted tumour suppressor role for Stat3 signalling in PCa by its ability to control the tumour suppressor ARF–Mdm2–p53 pathway in a context-dependent manner. Therefore, we propose the STAT3–ARF axis as a previously unknown safeguard mechanism against malignant progression in PCa. Importantly we also demonstrate that loss or inhibition of STAT3–ARF signalling enables tumour progression and metastasis formation. This is reflected in patients with PCa, since loss of STAT3 and/or p14^ARF^ expression significantly correlates with poor prognosis in large independent data sets. Therefore, our study identifies STAT3 and ARF as potential novel prognostic markers predicting BCR-free survival. The importance of the STAT3–ARF axis is corroborated by the presence of *STAT3* mutations in primary PCa and frequent deletions of *STAT3* and *ARF* in metastatic PCa. The significant loss of STAT3 and p14^ARF^ expression from matched primary and metastatic PCa samples underlines the relevance of our findings.

Interestingly, loss of PTEN expression in primary human PCa did not correlate with overall survival^52^ and could not predict PCa-specific death^53^. Moreover, heterozygous PTEN deletions far outnumber homozygous deletions in primary human PCa^54^ and we show here that PTEN is mutated or lost only in a small subset (4.7%) of a large cohort of patients with primary PCa. However, PTEN is lost in >50% of human PCa metastases^55^,^35^, suggesting an important role for PTEN in this process. Finally, we show in our study that STAT3 is co-deleted with PTEN in 66% of human PCa metastases in two independent data sets. Since PTEN is mutated or lost in only a minor fraction of primary PCa, other aberrations must occur (oncogene induction or loss of tumour suppressor function) to activate STAT3 and ARF to induce senescence in human cancers. Indeed, several studies indicate that different aberrations can lead to induction of senescence in human cancers^48^,^56^.

Many human PCa cases are diagnosed with low GSCs, which are of clinical low risk. Only a minority of these tumours will progress to aggressive lethal PCa. We show that ARF is an independent prognostic marker with a twofold higher hazard ratio compared with GSC (Fig. 7h). This will enable significantly improved stratification of low risk PCa patients into active surveillance, avoiding severe side effects such as incontinence and/or impotence. Together with a previously defined four-gene signature of aggressive tumours, including *PTEN*, *SMAD4*, *CCND1* and *SPP1* (ref. 16), determination of STAT3 and ARF expression could significantly improve the selection of patients with high risk PCa for personalized anti-cancer treatment.

We have uncovered a paradigm shift in understanding the key function of STAT3 in tumorigenicity and metastatic progression in PCa. Therefore, our results call for cautious use of anti-IL-6-STAT3 signalling blockers in the treatment of PCa as this may turn low-grade tumours into highly malignant cancers by loss of senescence controlled by the STAT3–ARF axis. As IL-6/STAT3 signalling blockers are successful in the treatment of chronic inflammatory or autoimmune diseases, their influence on PCa development needs to be carefully evaluated in future studies. Reactivating the IL-6/STAT3/ARF-dependent senescence pathway^57^ might be a promising strategy for PCa therapy via downregulation of Mdm2 (ref. 58) or p53 induction^59^. Alternatively, triggering ARF–p53-independent cellular senescence by a small molecule inhibitor^60^, could be beneficial for PCa patients in whom other therapies have failed.

## Methods

### Generation of transgenic mice

*Pten*^*loxP/loxP*^ mice crossed with male *PB-Cre4* transgenic mice were generated as described previously^13^,^61^. To generate prostate-specific deletion of *Pten* and *IL-6*, we took advantage of *IL-6*^*−/−*^ mice^30^. In a similar strategy, mice carrying *Stat3*^*loxP/loxP*^ 62 were maintained and crossed with *PB-Cre4 Pten*^*loxP/loxP*^ transgenic mice. All mice were maintained on a C57BL/6 and Sv/129 mixed genetic background. Animal experiments were reviewed and approved by the Austrian ministry authorities and conducted according to relevant regulatory standards (BMWF-66.009/0281-I/3b/2012).

### Immunohistochemistry and histological analysis

Immunohistochemistry and haematoxilin/eosin staining was performed with formalin-fixed paraffin-embedded (FFPE) tissue using standard protocols using consecutive sections. The following antibodies were used for immunohistochemistry: pY-Stat3 (1:80 dilution; Cell Signaling, #9145), pS-Stat3 (1:80; Cell Signaling, #9134), Stat3 (1:200 dilution; SCBT, sc-7179), p-Akt (1:80 dilution; Cell Signaling, #4060), Akt (1:100 dilution; Cell Signaling, #4691), p53 (1:50 dilution; Calbiochem, pAb421 ), p19^ARF^ (1:200 dilution; Abcam, ab-80), p14^ARF^ (1:100 dilution; SCBT, sc-8340), p21 (1:100 dilution; SCBT, sc-397), PML (1:100 dilution; SCBT, sc-966), Ki-67 (1:1,000 dilution; Novocastra; NCL-KI-67-P) and Cleaved Caspase 3 (1:200 dilution; Cell Signaling, #9661) and ISG15 (1:30; Abcam, 131119), AR (1:300; SCBT, sc-816). Staining for SA-ß-Gal activity was performed according to the manufacturer's protocol (Cell Signaling, #9860). We used PTEN^63^ and AR (1:250; DAKO, AR441)^64^ antibodies validated for FFPE IHC. The STAT3 antibody was validated using SW620 colon cancer xenografts with shRNA-mediated knockdown (Supplementary Fig. 9b). The specificity of p14^ARF^ antibody was validated using the human melanoma cell lines^65^ VM-28 (WT for p14^ARF^) and VM-7 (deletion at the p14^ARF^ locus).

All images were taken with a Zeiss AxioImager Z1, and quantification was performed with HistoQuest (TissueGnostics GmbH, Vienna, Austria, www.tissuegnostics.com) as described in detail in (ref. 36). In brief, haematoxylin staining was used for cell identification. The range of intensities of the master marker (haematoxylin) and the immunohistochemical stainings were set by autodetection of the software. All images were analysed with the identical settings after adjustments. The results are visualized in dot plot scattergrams and/or histograms. Cut-offs (to differentiate between positive and negative cells) and gates (to accentuate between cell populations) were set in the dot blots. For statistical analysis, the raw data were imported into GraphPad Prism 6 (GraphPad Software), analysed for significance and processed for data output. All images were taken with the same exposure time, signal amplification and objectives.

### Western blot analysis

For protein expression analysis by western blot, frozen tissue samples and cell lysates were prepared as described^16^. Blots were blocked with 5% BSA or 5% non-fat dry milk in 1 × TBS/0.1% Tween-20 for 1 h and incubated with the primary antibody overnight at 4 °C. Primary antibodies were reactive to pY-Stat3 (1:500 dilution), pS-Stat3 (1:1,000 dilution), Stat3 (1:1,000 dilution; Cell Signaling, #9132), p19^ARF^ (1:1,000 dilution), p53 (1:1,000 dilution; Cell Signaling, #2524), p21 (1:500 dilution), β-actin (1:5,000 dilution; Sigma-Aldrich, A5316), p-Akt (1:1,000 dilution), Akt (1:2,000 dilution ), AR (1:1,000 dilution), GAPDH (1:5,000 dilution; Cell Signaling, #2118), Pan-Cytokeratin (1,000 dilution; Abcam, Ab6401), Mdm2 (1:500 dilution; Millipore, 04-1530), PML (1:500 dilution; Millipore, MAB3738) and GAPDH (1:25,000; Trevigen, 2275-PC-100). Supplementary Figures 15–19 show uncropped immunoblots.

### RNA and qRT–PCR

Total RNA was isolated using Trizol (Invitrogen) according to the manufacturer's instructions. For quantitative reverse transcription PCR (qRT–PCR) analysis, 1 μg of total RNA was reverse transcribed to cDNA using the Transcriptor First-Strand cDNA Synthesis kit (Fermentas). qRT–PCR was performed in triplicate with aa MxPro3000 and SYBR GreenERqPCR mix (Invitrogen). Real-time monitoring of PCR amplification was performed using the LightCycler 480 detection system (Roche). The relative amount of specific mRNA was normalized to β-actin in each sample. Primer pairs are listed in Supplementary Table 3.

### Cell culture

Primary WT and *Stat3*-null MEF were isolated by trypsin treatment of individual littermate E13.5 embryos from a cross of *Stat3*^*+/−*^ heterozygous mice. *Stat3*^*+/−*^ mice were generated from conditional *Stat3*^*+/fl*^ mice^66^ by deletion of the conditional allele *in vivo* using *Mox2-Cre*. Cells were amplified and used in experiments starting at passage 2. *Stat3*^*−/−*^ MEFs were grown in DMEM supplemented with 10% FBS, 2 mM L-Glutamine, 0.1 mM NEAA, 20 mM HEPES and pen/strep using standard techniques. *Stat3*^*C/+*^ and *Stat3*^*C/C*^ MEFs were generously provided by Valeria Poli^28^. For *in vitro* cultures LNCaP, RWPE-1 and PC3 were cultured under standard conditions.

### Generation of primary mouse prostate cell lines

Dissected prostate tissue from WT and *Pten*^*pc−/−*^ mice at 19 weeks of age was cut into pieces of 0.2 mm, plated in tissue culture dishes coated with type I collagen (BD Pharmingen) and supplied with a minimal amount of epithelial growth medium containing 1% FBS serum. After 24 and 48 h, more medium was added. After an additional 24–72 h, cells began to grow out from the prostate tissue. Clean epithelial cell lines were obtained by removing the unwanted cell type with a cell scraper or pipette tip. Cells were passaged using Accutase (PAA, L11-00). For the generation of cell lines, cells were passaged until they survived crisis and only cells with typical epithelial morphology were maintained. WT and *Pten*^*pc−/−*^ prostate cell lines were established and maintained in DMEM/F12 (Invitrogen, 3133095) plus 10% foetal bovine serum (PAA, A15-101), 25 mg ml^−1^ bovine pituitary extract (Invitrogen, 13028014), 5 mg ml^−1^ EGF (Peprotech, 315-09), insulin/transferrin/selenite (Invitrogen, 41400-045), pen/strep (PAA, 30-002-CI), cholera toxin (Sigma-Aldrich, C8052-5MG) and ciprofloxacin (Sigma-Aldrich, 17580-5G). Prostate tumour epithelial cell lines expressed the epithelial marker Pan-Cytokeratin detected by immunofluorescence and western blot analysis. *Pten* deletion was confirmed by PCR and western blot analysis.

### Human tissue microarray

The human tissue arrays representing a panel of 204 patient samples were obtained from the Institute of Pathology, Tuebingen, Germany and from the Clinical Institute of Pathology (CIP) of the Medical University of Vienna (MUW), Vienna, Austria. The cohort contains tumour material from PCa patients who consecutively underwent radical prostatectomy at the University Hospital of Tuebingen or the University Hospital of the MUW. Each PCa specimen was represented by two cores on tissue microarrays (TMA). From the CIP of the MUW, we furthermore obtained 41 PCa and 23 metastasis FFPE patient samples. In addition, we include FFPE material from 67 matched patient samples with PIN and PCa (GSC≤7), as well as 7 matched patient samples with primary PCa and PCa metastases, all from the CIP, MUW. The FFPE tissue blocks were sectioned as 3-μm-thick sections mounted on slides and stained with haematoxylin and eosin. Subsequently, the area of cancer was marked by a pathologist (S.P.). Cores, each of up to 0.6 mm in diameter, were taken from the corresponding donor block and placed into a TMA recipient block using a semiautomatic tissue arrayer (Beecher Instruments). Tissue sections (3-μm thick) were placed onto superfrost slides. IBM SPSS Statistics 20 (IBM) was used for statistical analysis. For determination of time to BCR, which is defined by an increase to >0.2 ng ml^−1^ PSA in serum, a log-rank (Mantel–Cox) test was used to test significance. The hazard ratio and its confidence interval were calculated using the Mantel–Haenszel method. All the human samples for TMA and their use in this study were approved by the Research Ethics Committee of the Medical University Vienna, Austria (1753/2014) and by the Research Ethics Committee for Germany (395/2008BO1) (Bonn, Germany).

### Statistical analyses

Data were analysed using GraphPad Prism 6 software. For comparing two groups unpaired Students *t*-test and for comparing more than two groups Tukeýs *post hoc* test was used. Fisher's exact test was employed when differences in distributions within groups were monitored. For Kaplan–Meier analysis and log-rank statistical evaluation of time to BCR, as well as evaluation of prognostic power in univariate and multivariate analysis, we used the IBM SPSS version 22 programme. To analyse Affymetrix SNP Chip 6.0 primary data, we used the AGCCytoScan Software together with the Chromosome Analysis Suite 3.0 (both Affymetrix), and deletions >200 kb were considered significant. For analysis of mutations in primary PCa, we used the ICGC (www.icgc.org).

### ELISA

For the detection of soluble sIL-6R in sera of mice, the samples were diluted 1:15 in 1% BSA–PBS. The ELISA was performed as described^67^. In brief, microtiter plates (Greiner Microlon) were coated with anti-murine IL-6-R pAB AF1830 (0.8 μg ml^−1^; R&D Systems) in PBS. After blocking, 100 μl aliquots of cell lysates or culture supernatants were added. IL-6R bound to the plate was detected by biotinylated goat anti-mouse IL-6-R pAB BAF1830 (working concentration, 0.2 μg ml^−1^; R&D Systems) followed by streptavidin–horseradish peroxidase (R&D Systems). The enzymatic reaction was performed with soluble peroxidase substrate (BM blue POD from Roche) at 37 °C and the absorbance was read at 450 nm on a SLT Rainbow plate reader (Tecan). For the detection of murine IL-6 in sera of mice, the samples were diluted 1:3 with 1% BSA–PBS.

## Additional information

**How to cite this article:** Pencik, J. *et al.* STAT3 regulated ARF expression suppresses prostate cancer metastasis. *Nat. Commun.* 6:7736 doi: 10.1038/ncomms8736 (2015).

## Supplementary Material

Supplementary Information Figures 1-19, Supplementary Tables 1-3, Supplementary Methods and Supplementary References
