# Erratum: STAT3 regulated ARF expression suppresses prostate cancer metastasis

**DOI:** 10.1038/ncomms9802

**Published:** 2015-10-26

**Authors:** Jan Pencik, Michaela Schlederer, Wolfgang Gruber, Christine Unger, Steven M. Walker, Athena Chalaris, Isabelle J. Marié, Melanie R. Hassler, Tahereh Javaheri, Osman Aksoy, Jaine K. Blayney, Nicole Prutsch, Anna Skucha, Merima Herac, Oliver H. Krämer, Peter Mazal, Florian Grebien, Gerda Egger, Valeria Poli, Wolfgang Mikulits, Robert Eferl, Harald Esterbauer, Richard Kennedy, Falko Fend, Marcus Scharpf, Martin Braun, Sven Perner, David E. Levy, Tim Malcolm, Suzanne D. Turner, Andrea Haitel, Martin Susani, Ali Moazzami, Stefan Rose-John, Fritz Aberger, Olaf Merkel, Richard Moriggl, Zoran Culig, Helmut Dolznig, Lukas Kenner

10.1038/ncomms8736

The affiliation details for Jan Pencik are incorrect in this Article. The correct affiliation details for this author are given below:

Ludwig Boltzmann Institute for Cancer Research, Waehringerstrasse 13A, 1090 Vienna, Austria.

Clinical Institute of Pathology, Medical University of Vienna, 1090 Vienna, Austria.

