# Supplementary material for: STAT3 regulated ARF expression suppresses prostate cancer metastasis
Source: Nat Commun. 2015 Jul 22;6:7736. doi: 10.1038/ncomms8736 (PMC4525303; doi:10.1038/ncomms8736)
Supplement: Supplementary Information — Figures 1-19, Supplementary Tables 1-3, Supplementary Methods and Supplementary References [file ncomms8736-s1.pdf]

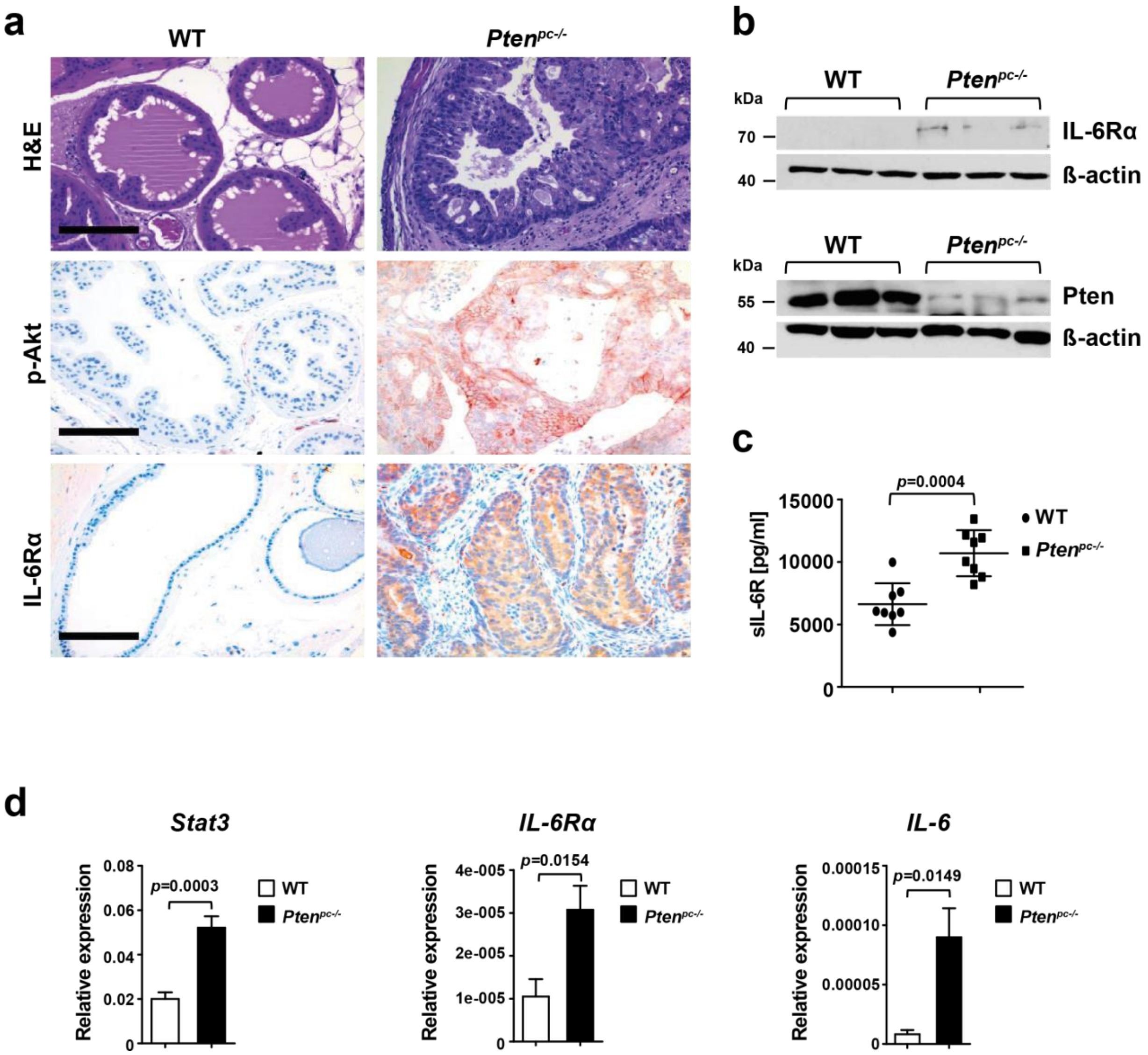

Supplementary Figure 1: Pten loss induces the IL-6/IL-6Rα signaling pathway.

a, Comparison of H&E and IHC analysis of p-Akt and IL-6Rα of prostates from WT and Pten-deficient 19-week old mice. Scale bars, 100 µm. **b,** Pten and IL-6α protein expression determined by immunoblotting in 19-week old WT and *Pten*$^{pc-/-}$ prostates. β-actin serves as loading control. **c,** Plasma levels of soluble IL-6R in WT and *Pten*$^{pc-/-}$ prostates (19-week old mice); *n*≤10 **d,** qRT-PCR analysis to determine mRNA expression of *Stat3*, *IL-6Rα* and *IL-6* genes in WT and *Pten*$^{pc-/-}$ prostates. Mean values are shown; error bars: s.d. (*n*=5). Data from (c) and (d) were analysed by Student's t-test.

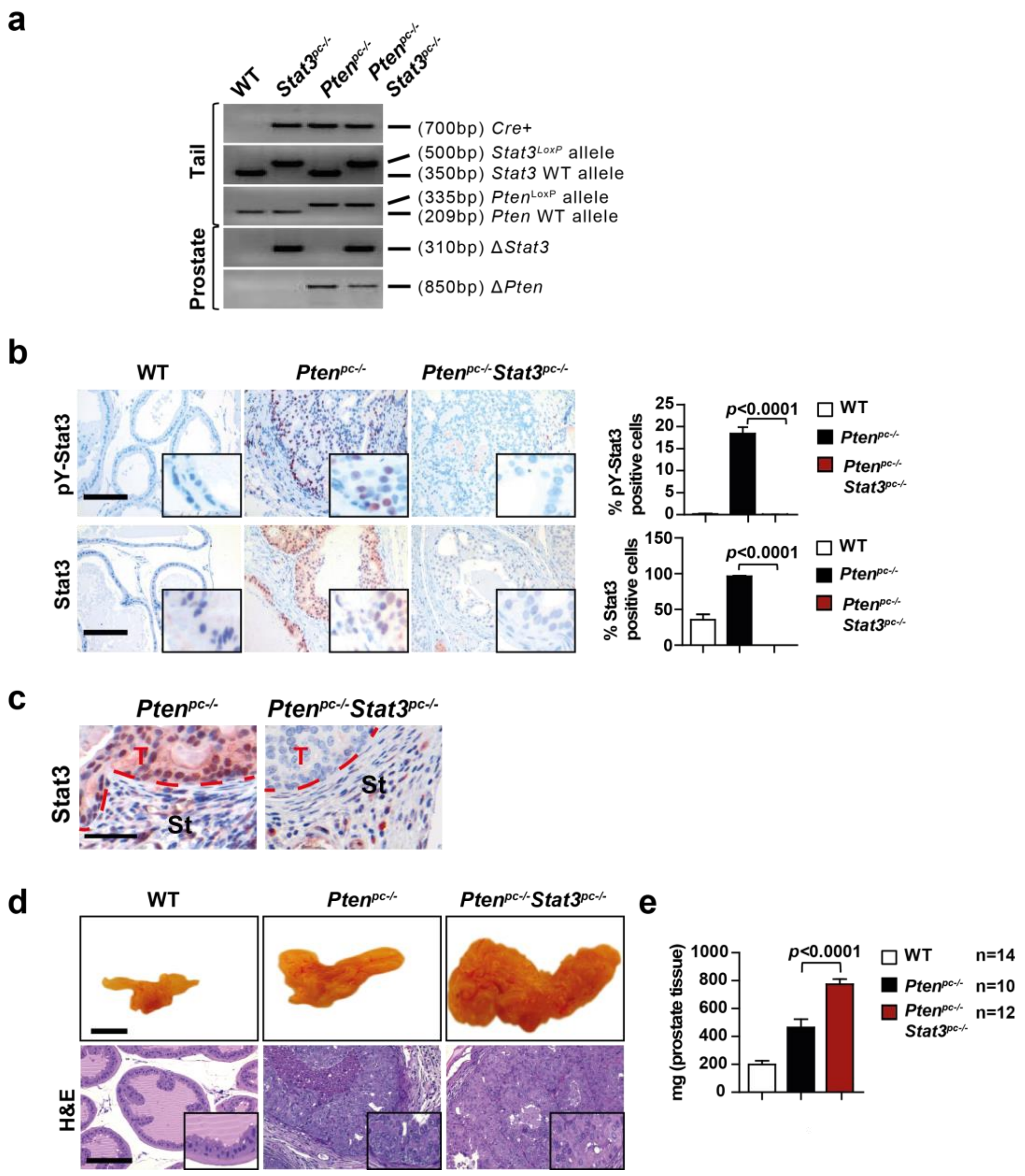

Supplementary Figure 2: Acceleration of PCa development in *Pten*$^{pc-/-}$*Stat3*$^{pc-/-}$ mice.

a, PCR-based identification of mutants from non-recombined and recombined allelic combinations of *Pten*^{loxP} *and Stat3*^{loxP} and alleles prior to Cre-mediated recombination on tail DNA, or after Cre-mediated recombination in purified genomic DNA from prostates. **b,** IHC analysis of pY-Stat3 and Stat3 in prostates from 19-week old WT, *Pten*$^{pc-/-}$ and *Pten*$^{pc-/-}$*Stat3*$^{pc-/-}$ mice. Scale bars, 100 µm. pY-Stat3 and Stat3 quantification was done with HistoQuest^{TM} software. Mean values are shown; error bars: s.d. $p < 0.0001$ (n=5). Data from were analysed by Student's t-test. **c,** Representative Stat3 IHC of a mouse prostate adenocarcinoma at 19 weeks of age. Dashed red line indicates the border of tumor (T) to stroma (St). Scale bars, 100 µm. **d,** Gross anatomy of representative prostates at 19 weeks p.p. from WT, *Pten*$^{pc-/-}$ and *Pten*$^{pc-/-}$*Stat3*$^{pc-/-}$ mice. Scale bars, 10 mm. H&E sections show a poorly differentiated, highly invasive PCa in *Pten*$^{pc-/-}$*Stat3*$^{pc-/-}$ mice compared to well differentiated PCa in *Pten*$^{pc-/-}$ mice. Scale bars, 100 µm. **e,** Weights of prostates or PCa of 19-week old WT, *Pten*$^{pc-/-}$ and *Pten*$^{pc-/-}$*Stat3*$^{pc-/-}$ mice (n = 32). Mean values are shown; error bars: s.d. Data from (b) and (e) were analysed by Student's t-test.

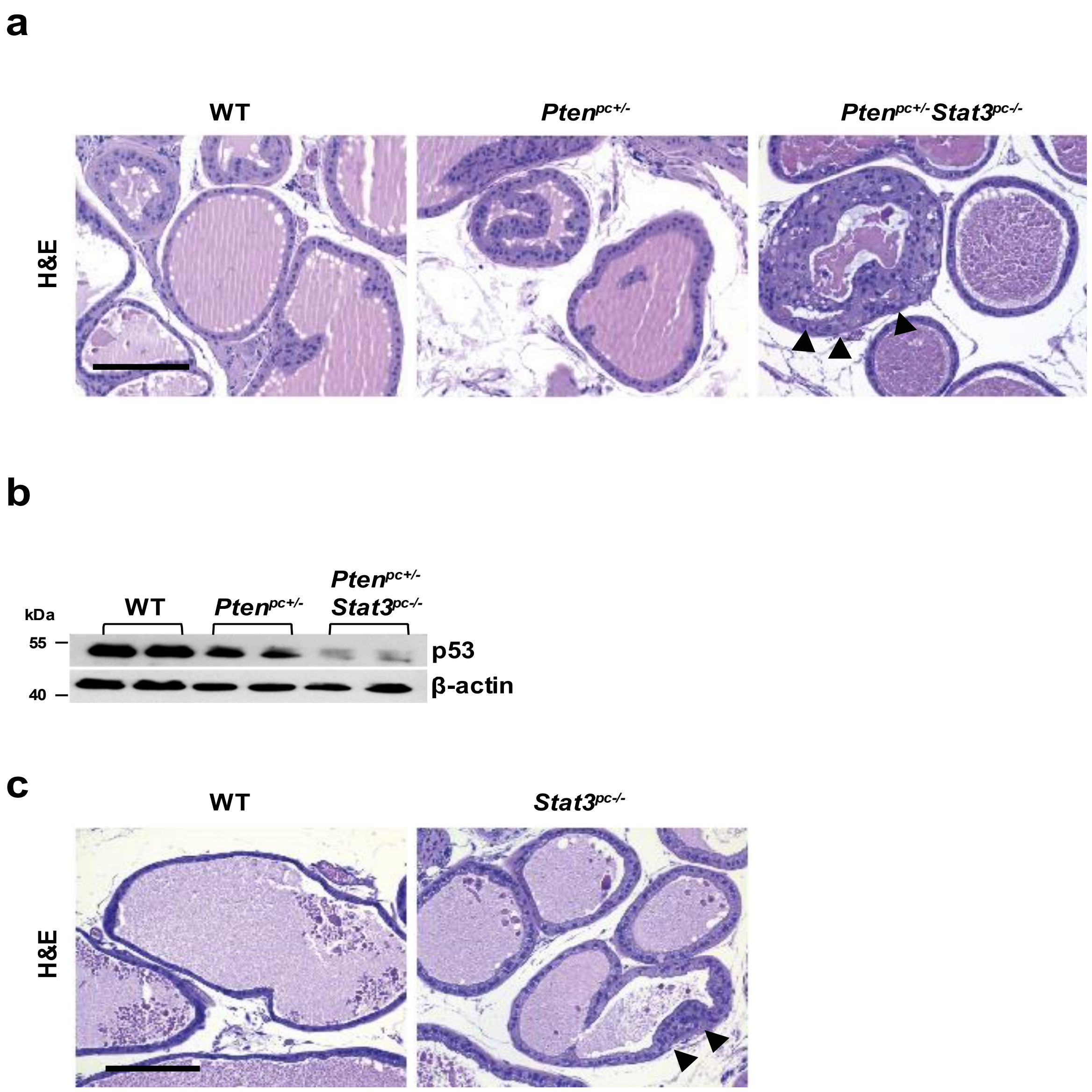

Supplementary Figure 3: *Stat3* inactivation cooperates with *Pten* haploinsufficiency to promote PCa development.

a, H&E analysis of prostates from WT, *Pten*$^{pc+/-}$, and *Pten*$^{pc+/-}$*Stat3*$^{pc-/-}$ mice at 19 weeks of age. Arrowheads point to the *in situ* adenocarcinoma. Scale bars, 100 µm. **b,** Western blot analysis of p53 levels in prostates from WT, *Pten*$^{pc+/-}$, and *Pten*$^{pc+/-}$*Stat3*$^{pc-/-}$ mice. **c,** H&E analysis of WT and *Stat3*$^{pc-/-}$ prostates from 19-week old mice. Arrowheads indicate prostatic intraepithelial neoplasia (PIN). Scale bars, 100 µm.

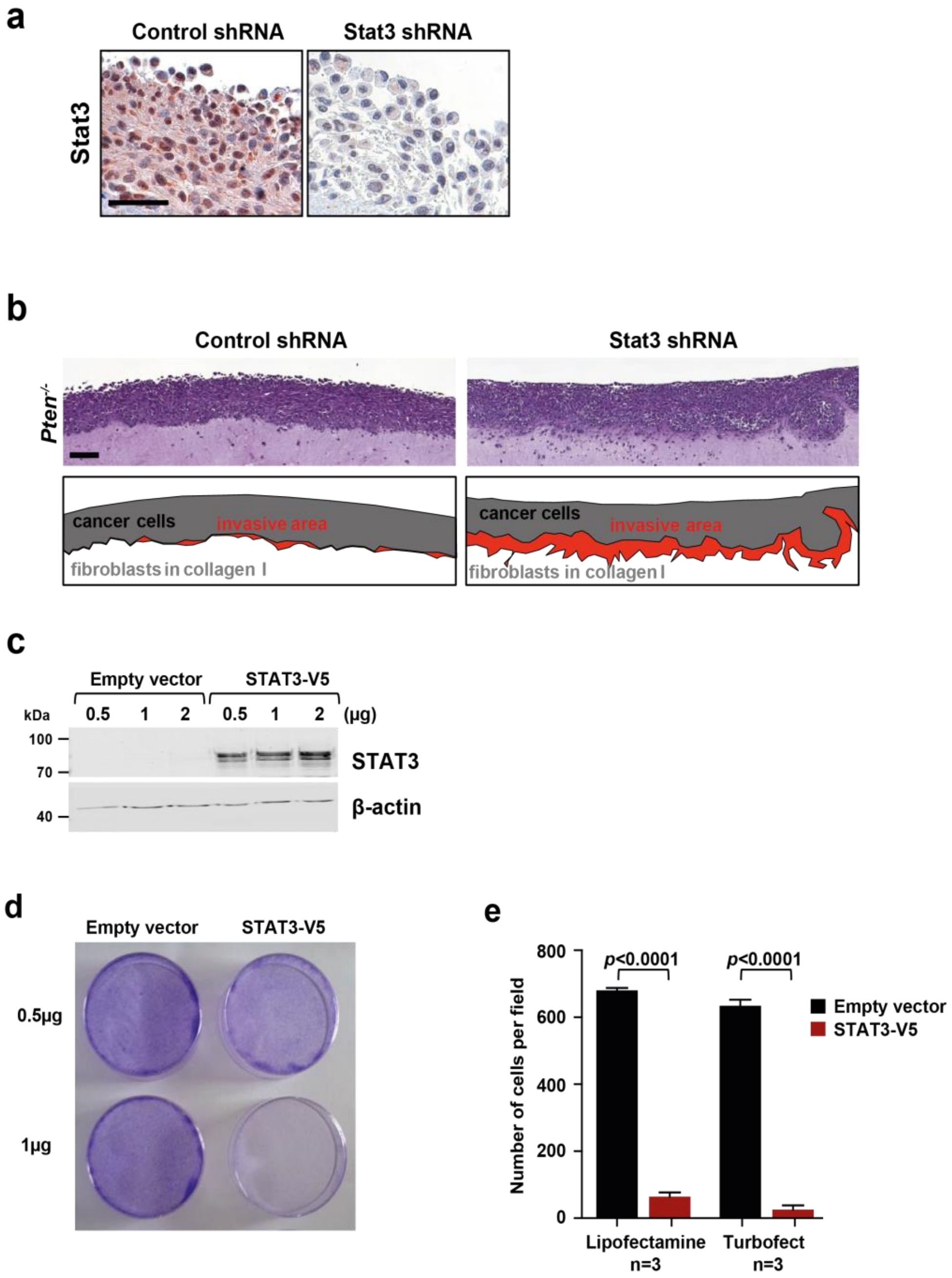

Supplementary Figure 4: Loss of STAT3 and PTEN significantly enhanced invasion of prostate cells.

a, Efficient knockdown of Stat3 was demonstrated by Stat3 IHC analysis in sections of the organotypic assays shown in Fig. 3c. Scale bar, 100 µm. **b,** Organotypic culture assays of *Pten*$^{-/-}$ mouse PCa cells in the presence and absence of Stat3 cultivated in contact with mouse prostate stromal fibroblasts, fixed in formalin, embedded in paraffin and stained with H&E after 8 days. Scale bars, 100 µm. **c**, Western blot analysis of PC3 cells transfected with empty vector (0.5-2 µg) or STAT3-V5 (0.5-2 µg). Antibodies against STAT3 or β-actin serve as loading control. **d**, Crystal violet stained PC3 cells transfected with empty vector or STAT3-V5. **e**, Cell numbers of PC3 (n=4) 24 hours after transfection with empty vector or STAT3-V5 seeded at equal densities prior to transfection transfection either by Lipofectamin (n=3) or Turbofect (n=3). Bars indicate mean cell numbers, p < 0.0001. Mean values are shown; error bars: s.d. Data from (e) were analysed by Student's t-test.

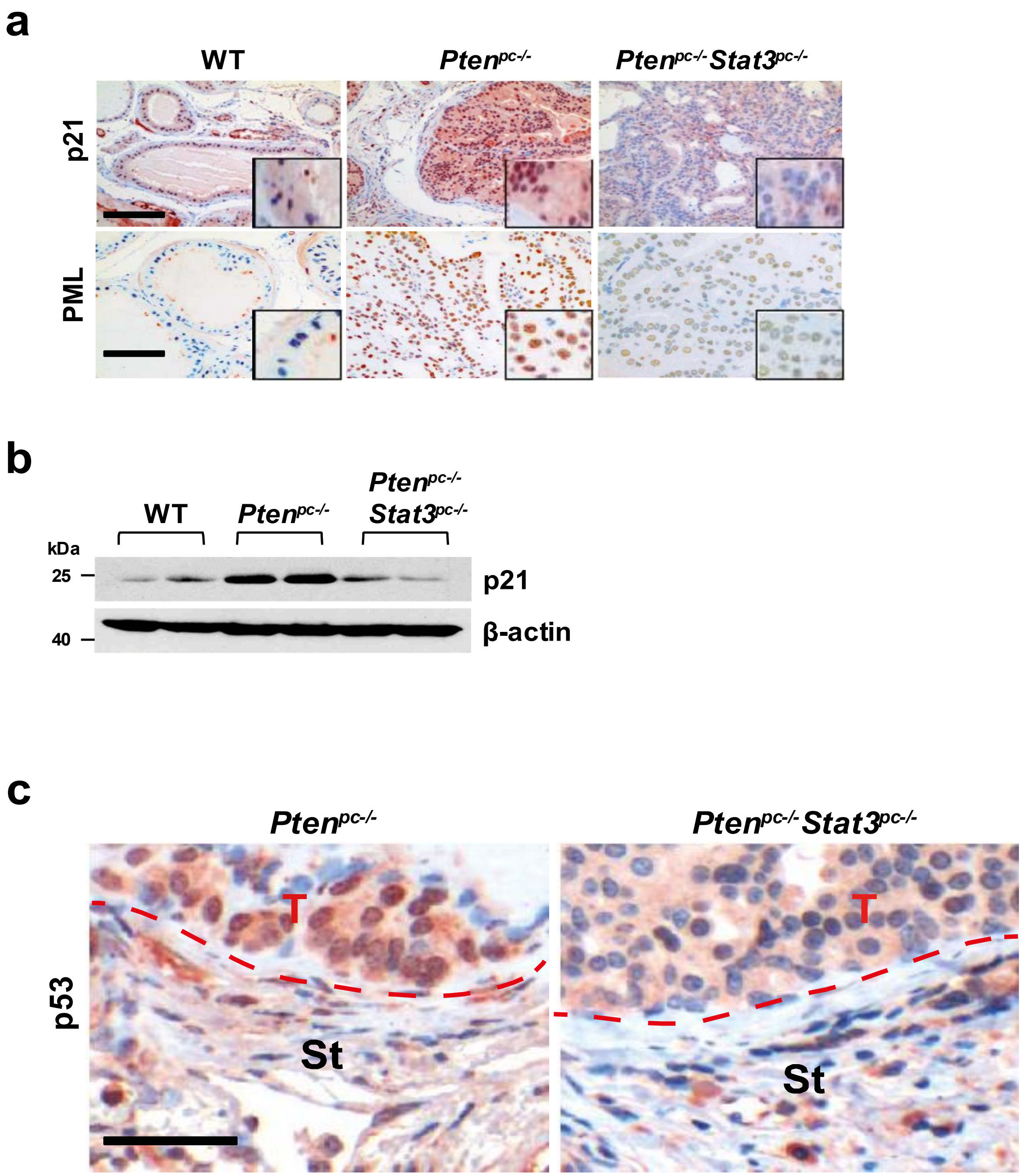

Supplementary Figure 5: Decreased senescence markers in PCa of *Pten*$^{pc-/-}$ *Stat3*$^{pc-/-}$ mice.

a, IHC analysis of p21 and PML in prostates from 19-week old WT, *Pten*$^{pc-/-}$ and *Pten*$^{pc-/-}$*Stat3*$^{pc-/-}$ mice. Scale bars, 100 µm. **b,** Western blot analysis of p21 in WT, *Pten*$^{pc-/-}$ and *Pten*$^{pc-/-}$*Stat3*$^{pc-/-}$ prostate tissue. **c,** Representative images of p53 IHC of *Pten*$^{pc-/-}$ and *Pten*$^{pc-/-}$*Stat3*$^{pc-/-}$ mouse prostate adenocarcinomas at 19 weeks p.p. are shown. Dashed red line indicates the border of tumor (T) to stroma (ST). Scale bars, 100 µm.

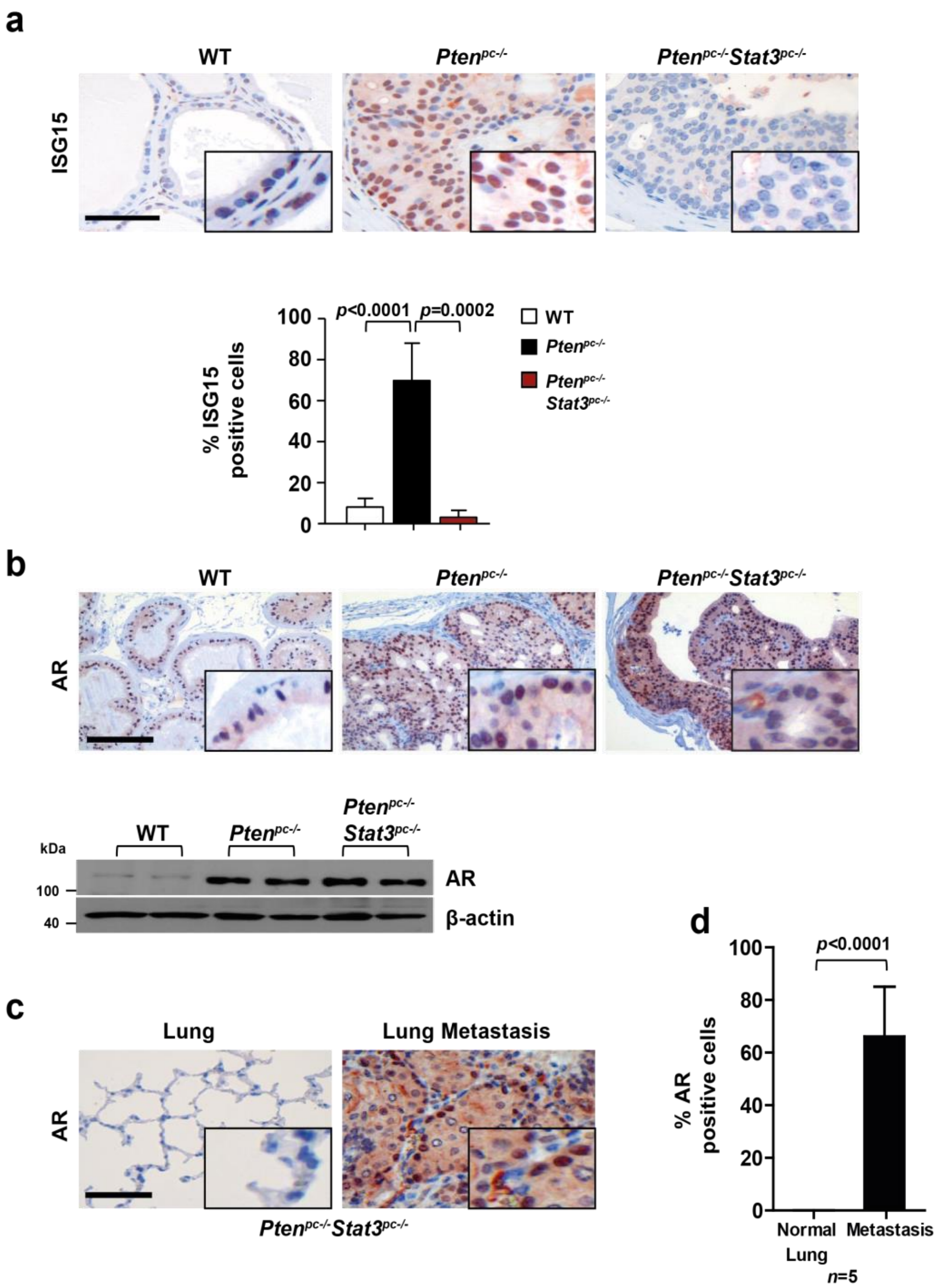

Supplementary Figure 6: ISG15 and AR expression in *Pten*$^{pc-/-}$*Stat3*$^{pc-/-}$ PCa.

a, IHC analysis of ISG15 expression in prostates from 19-week old WT, *Pten*$^{pc-/-}$ and *Pten*$^{pc-/-}$*Stat3*$^{pc-/-}$ mice. Scale bars, 100 µm. ISG15 quantification was done with HistoQuest^{TM} software. Bar graphs indicate percentage of ISG15 positive cells. (n=5). **b,** IHC analysis of androgen receptor (AR) in prostates from 19-week old WT, *Pten*$^{pc-/-}$ and *Pten*$^{pc-/-}$*Stat3*$^{pc-/-}$ mice. Scale bars, 100 µm. Western blot analysis of AR in WT, *Pten*$^{pc-/-}$ and *Pten*$^{pc-/-}$*Stat3*$^{pc-/-}$ prostate tissue. **c,** IHC staining for AR of a representative murine lung metastasis and surrounding normal lung of *Pten*$^{pc-/-}$*Stat3*$^{pc-/-}$ mice at 52 weeks of age. Scale bar, 100 µm. **d,** Quantification of AR staining in 52-week old *Pten*$^{pc-/-}$ *Stat3*$^{pc-/-}$ lung metastases using HistoQuest^{TM} software, p < 0.0001. Mean values are shown; error bars: s.d. (n=5). Data from (a) and (d) were analysed by Student's t-test.

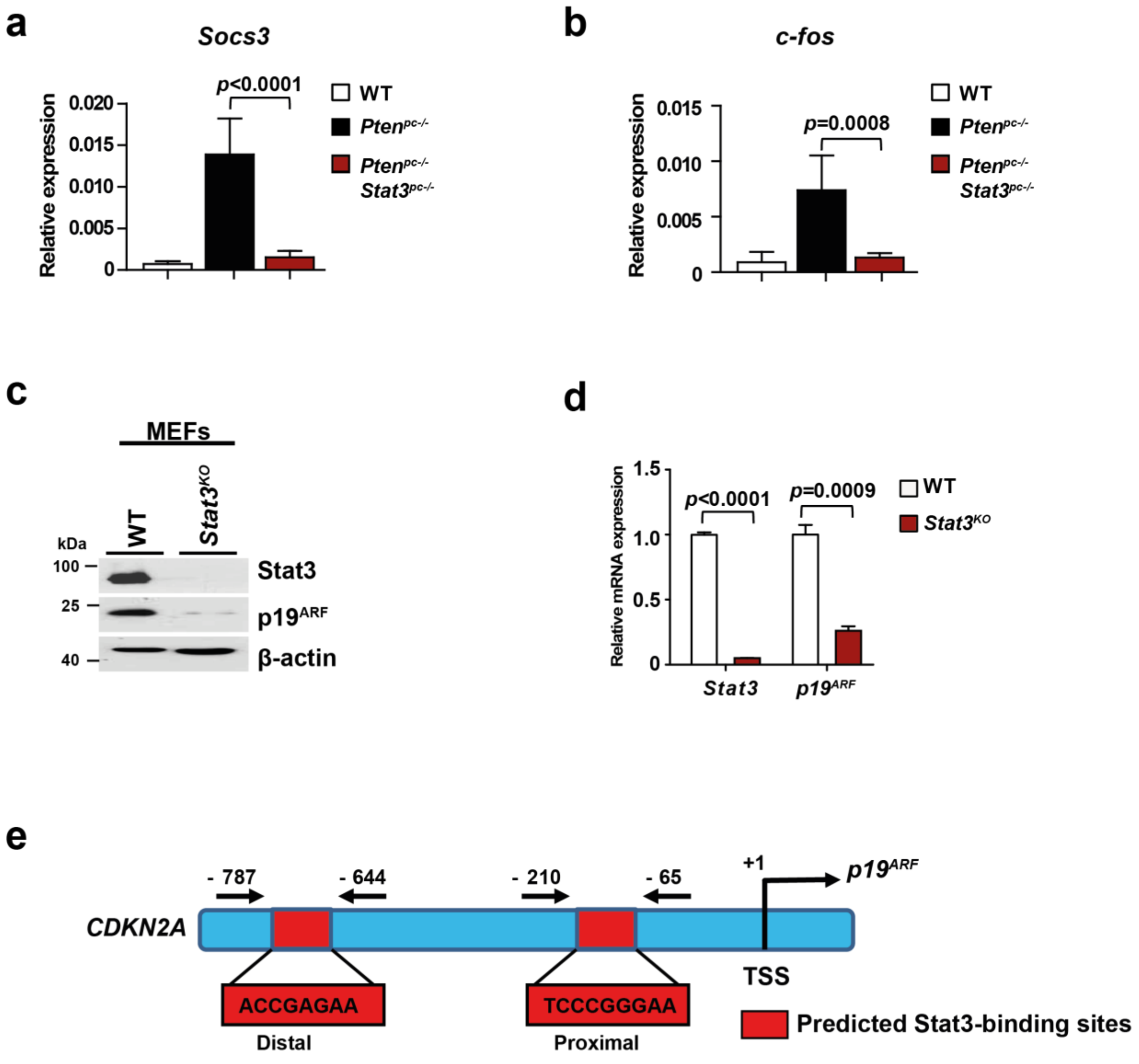

Supplementary Figure 7: Analysis of Stat3 target genes.

qRT-PCR analysis of **a,** *Socs3* and **b,** *c-fos* mRNA expression in prostates of 19-week old WT, *Pten*$^{pc-/-}$ and *Pten*$^{pc-/-}$*Stat3*$^{pc-/-}$ mice. Mean values are shown; error bars: s.d. (n = 5). **c,** Western blot analysis of Stat3 and p19^{ARF} expression in WT and *Stat3* knockout (*Stat3*^{KO}) mouse embryonic fibroblasts (MEFs). **d,** qRT-PCR analysis of *Stat3* and *p19*^{ARF} transcript levels in WT and *Stat3*^{KO} MEFs (n = 3). **e,** Schematic representation of the *CDKN2A* locus including the *p19*^{ARF} promoter. *In silico* predicted proximal and distal Stat3 binding sites are indicated in red. Data from (a), (b) and (d) were analysed by Student's t-test.

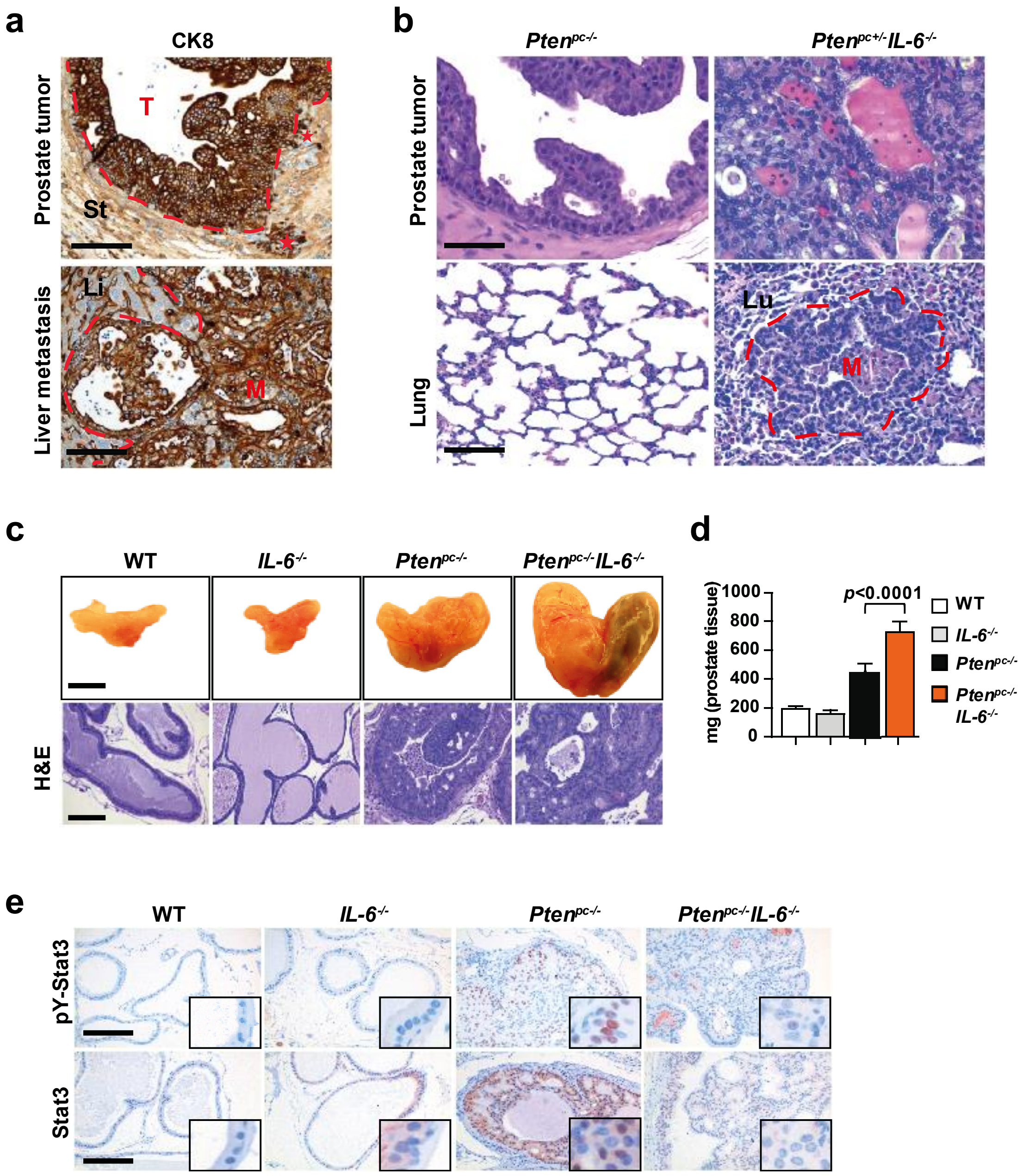

Supplementary Figure 8: Loss of IL-6 in *Pten*$^{pc-/-}$ mice enhances tumorigenesis and leads to distant liver and lung metastasis of PCa.

a, Cytokeratin 8 (CK8) IHC staining of primary PCa and liver metastasis of *Pten*$^{pc-/-}$*IL-6*$^{-/-}$ mice at 38 weeks of age. Dashed red lines indicate the border of tumor (T) to stroma (St) and of metastasis (M) to normal liver (Li). Scale bar, 150 µm **b,** H&E analysis of prostate tumor and lung from *Pten*$^{pc-/-}$ and *Pten*$^{pc+/-}$*IL-6*$^{-/-}$ mice at 60 weeks of age. The dashed red line encircles the border of an advanced lung metastasis (M) to normal lung (Lu) in *Pten*$^{pc+/-}$*IL-6*$^{-/-}$ mice. Scale bar, 100 µm. **c,** Gross anatomy of representative prostates at 19 weeks of age from WT, *IL-6*$^{-/-}$, *Pten*$^{pc-/-}$ and *Pten*$^{pc-/-}$*IL-6*$^{-/-}$ *mice*. Scale bar, 10 mm. H&E stained sections of *Pten*$^{pc-/-}$*IL-6*$^{-/-}$ prostates show poorly differentiated PCa with accelerated progression compared to well differentiated PCa in *Pten*$^{pc-/-}$ prostates. WT and *IL-6*$^{-/-}$ mice serve as controls. Scale bar, 100 µm **d,** Prostate weights of 19 weeks old WT, *IL-6*$^{-/-}$, *Pten*$^{pc-/-}$ and *Pten*$^{pc-/-}$*IL-6*$^{-/-}$ mice. Mean values are shown; error bar: s.d. (*n*=10). **e,** IHC analysis of pY-Stat3 and Stat3 in prostates from 19-week old WT, *IL-6*$^{-/-}$, *Pten*$^{pc-/-}$ and *Pten*$^{pc-/-}$*IL-6*$^{-/-}$ mice. Scale bars, 100 µm. Data from (d) were analysed by Student's t-test.

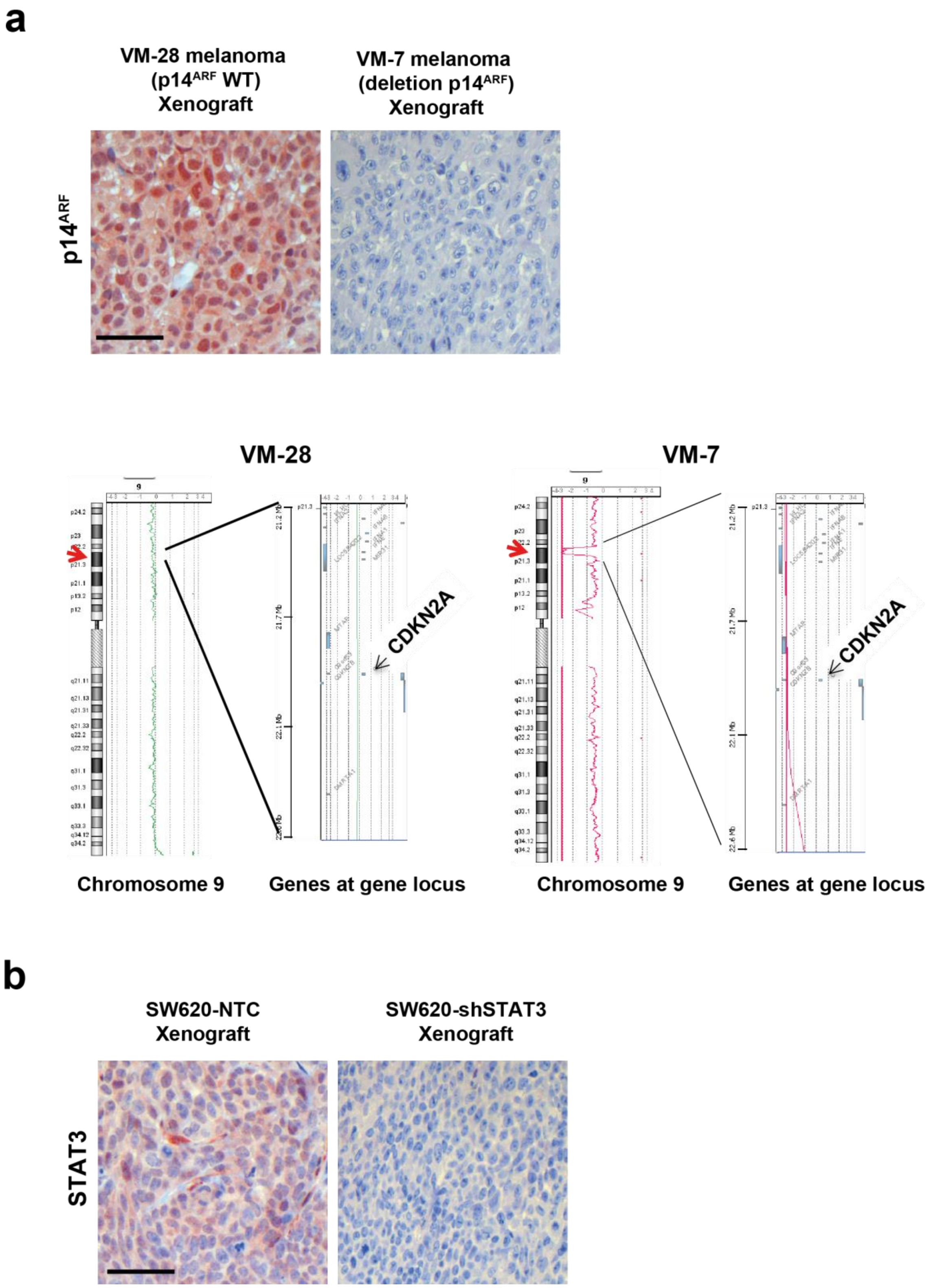

Supplementary Figure 9: p14^{ARF} and STAT3 antibodies evaluation for IHC.

a, b IHC evaluation of p14^{ARF} and STAT3 antibodies under the same conditions as for TMA analysis using human xenograft tumors from VM-28 (control) or VM-28 (p14^{ARF} deleted) melanoma cell lines or SW620 (with and without shSTAT3) colon cancer cell lines, respectively. Scale bars, 100 µm.

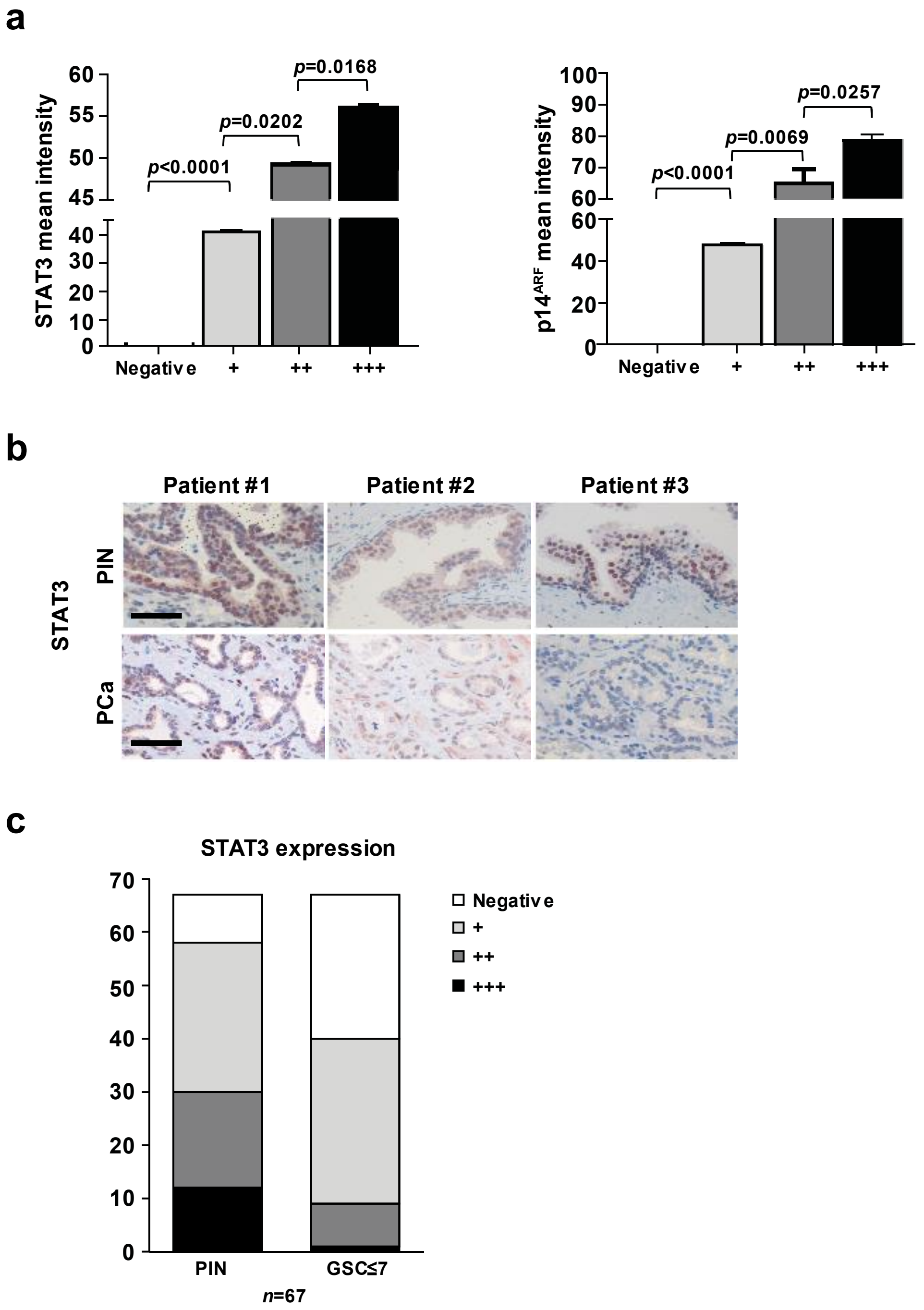

Supplementary Figure 10: Expression and clinical relevance of STAT3 and p14^{ARF} are assessed in a human PCa TMA.

a, The STAT3 or p14^{ARF} protein expression levels are subdivided into Negative, +, ++ and +++ positive tissues. Analysis of primary PCa (*n*=204) TMAs from patients after radical prostatectomy was carried out independently by board certified pathologists. The quantification of STAT3 and p14^{ARF} stainings was validated by using the HistoQuest^{TM} software. Mean intensities are shown, error bars depict s.d. Data in (a) were analysed by one-way ANOVA with Tukey's multiple comparison test. **b,** Three representative images of STAT3 expression in matched patients samples PIN vs primary PCa (GSC≤7) are shown (*n*=67). Scale bars, 100 µm. **c,** The quantification of STAT3 analysis described in (b), was validated by using the HistoQuest^{TM} software. Mean intensities are shown, error bars depict s.d. (*n*=67).

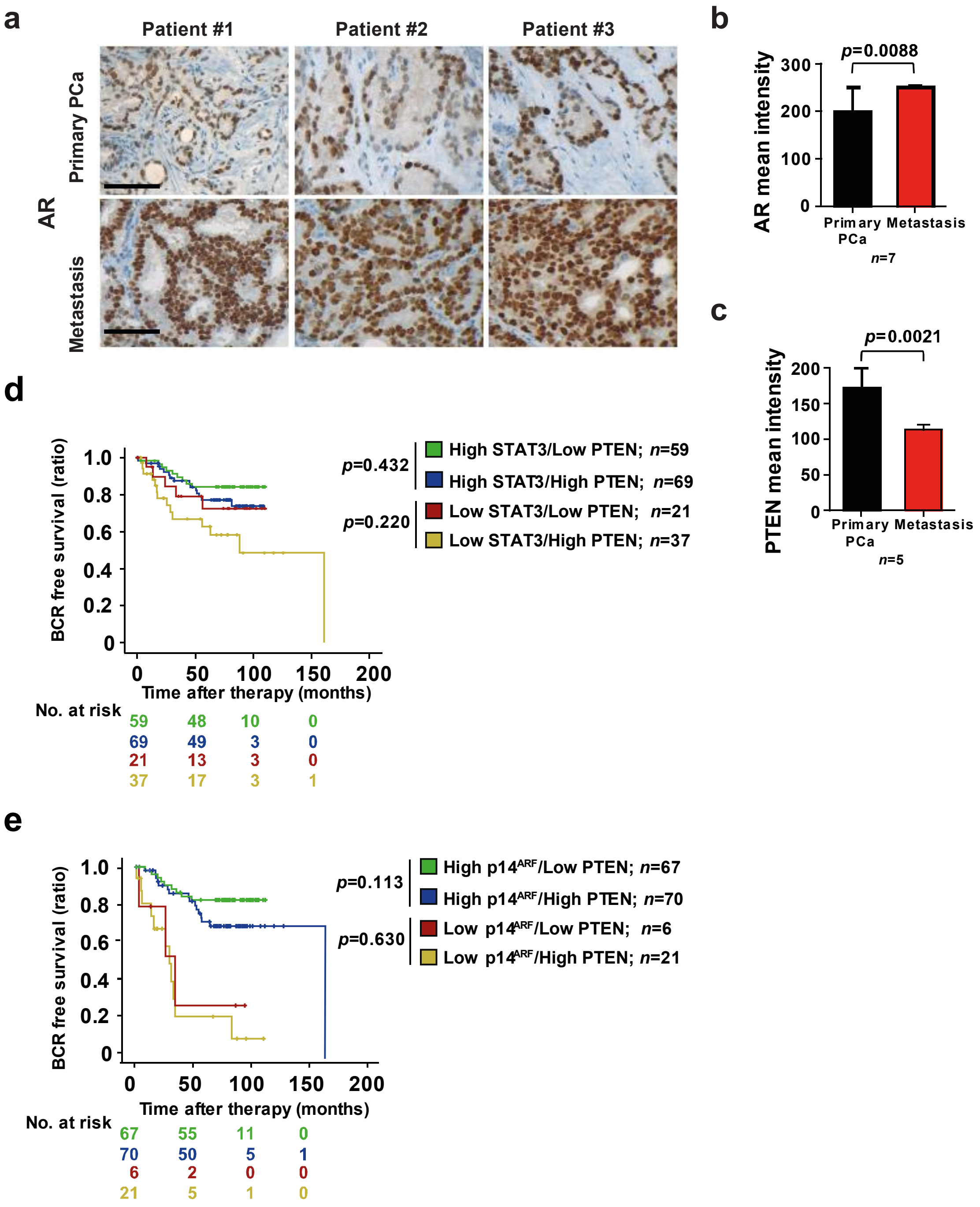

Supplementary Figure 11: Loss of STAT3 and/or p14^{ARF} is not associated with loss of PTEN.

a, Three representative images of AR expression determined by IHC analyses in matched patient samples with primary and metastatic PCa (*n*=7). Scale bars, 100 µm. **b** Bar graphs indicate mean intensity of AR expression determined by IHC analyses in matched patient samples with primary and metastatic PCa; error bars depict s.d. (*n*=7). **c** Bar graphs indicate mean intensity of PTEN expression determined by IHC analyses in matched patient samples with primary and metastatic PCa; error bars depict s.d. (*n*=5). Data from b and c were analysed by Student's t-test. **d,** Kaplan–Meier analysis of BCR-free survival ratio based on STAT3 and PTEN protein expression (log-rank test) in a panel of 204 PCa patients. **e**, Kaplan–Meier analysis of BCR-free survival ratio based on p14^{ARF} and PTEN protein expression (log-rank test) in a panel of 204 PCa patients. No. at risk are shown in d, e.

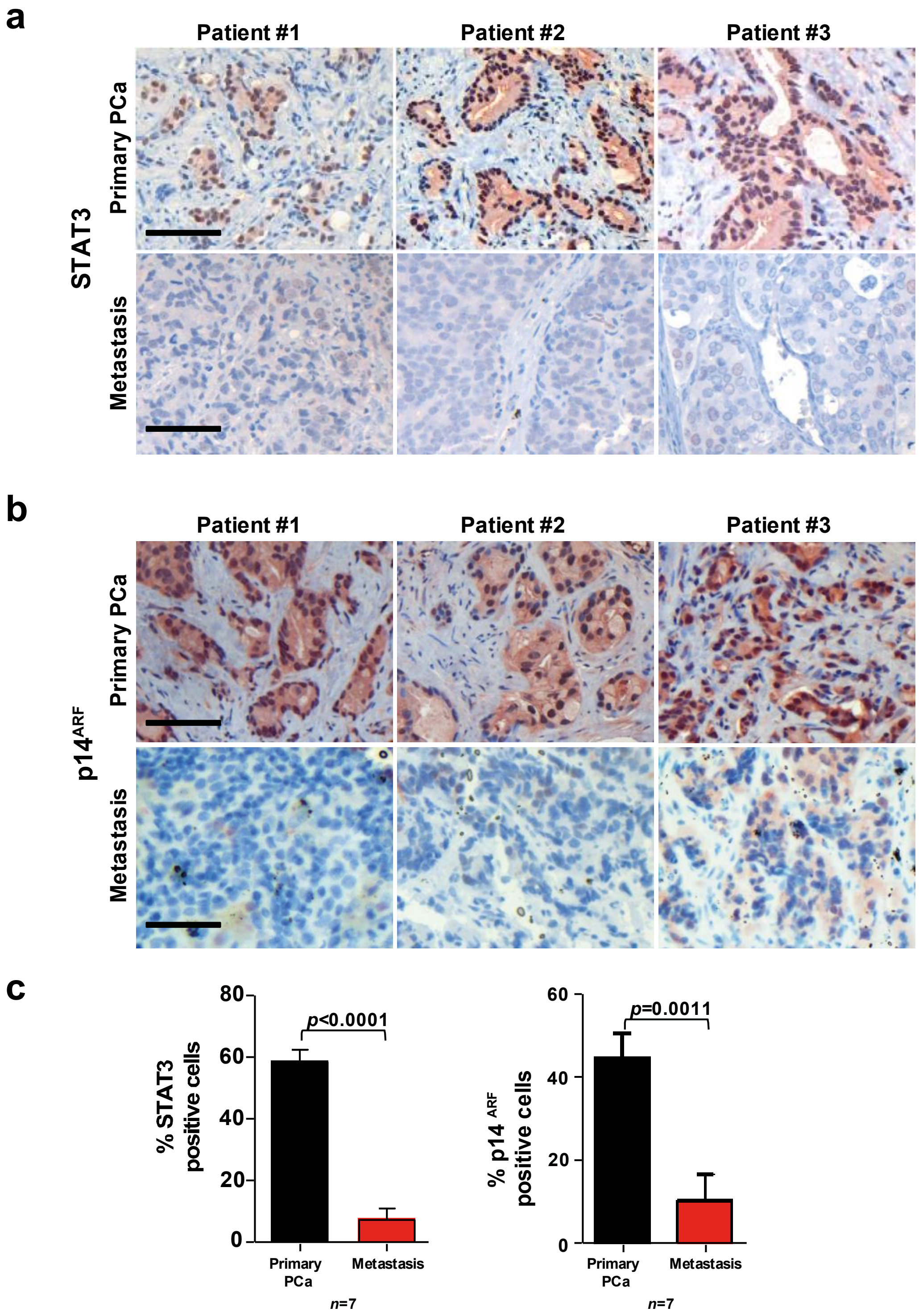

Supplementary Figure 12: Loss of STAT3 and p14^{ARF} protein expression robustly correlates with metastatic PCa in matched patient samples.

a, **b,** Three representative images of STAT3 and p14^{ARF} expression determined by IHC in matched patient samples with primary and metastatic PCa (*n*=7). Scale bars, 100 µm. **c**, Quantitative image analysis of STAT3 and p14^{ARF} expression in patient samples described in (a, b) using the HistoQuest^{TM} software. Bar graphs indicate percentage of cells positive for STAT3 (b) and p14^{ARF} (c), error bars depict s.d. (*n*=7). Data from (a) and (b) were analysed by Student's t-test.

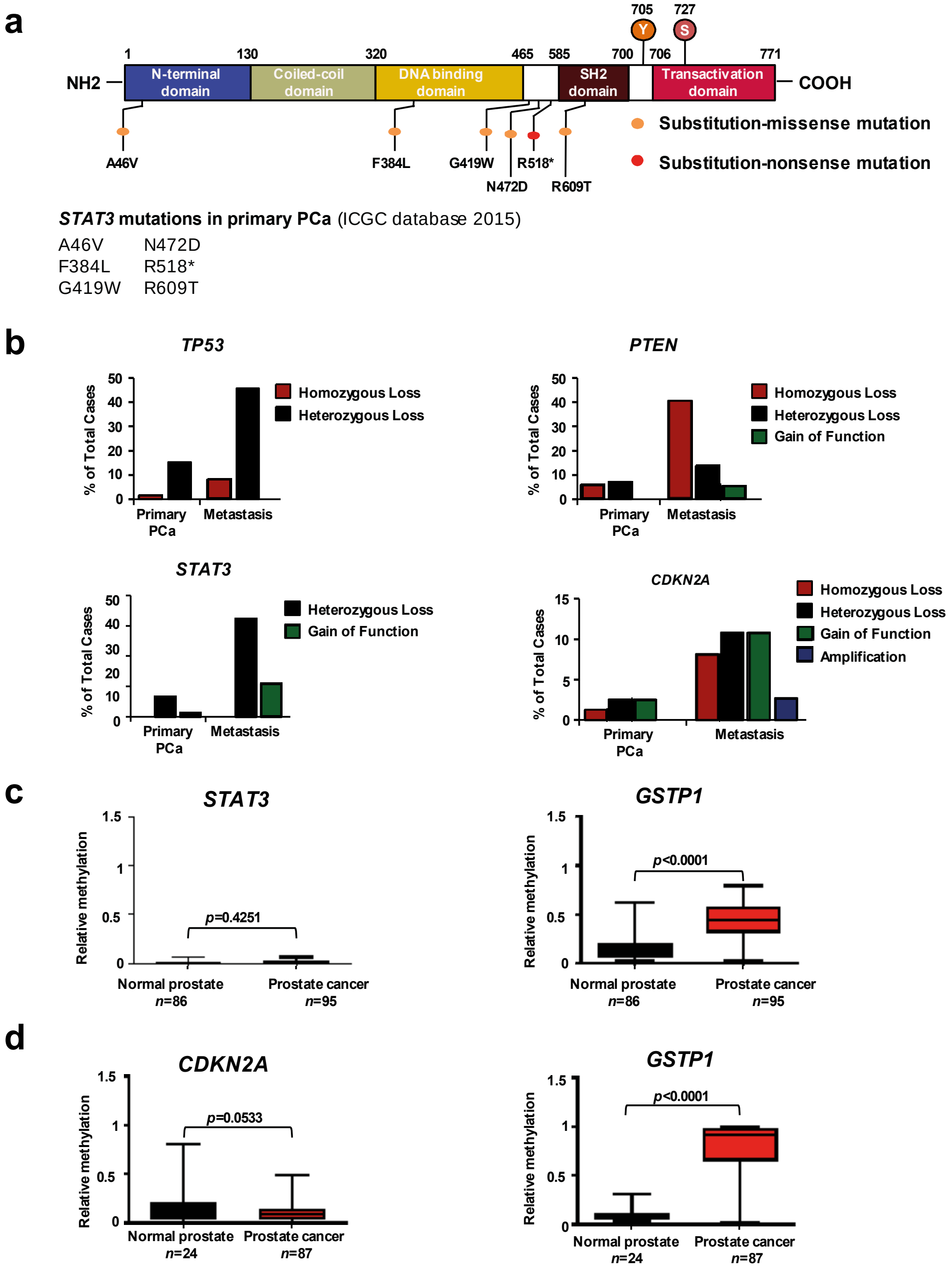

Supplementary Figure 13: *STAT3* and *CDKN2A* mutations define a distinct genetic subclass of aggressive PCa.

a, Graphic representation of *STAT3* mutations in primary PCa (ICGC database 2015). **b**, Analysis of *TP53*, *PTEN*, *STAT3* and *CDKN2A* genetic aberrations in 243 primary PCa compared to 37 metastases. Percentages of total cases are shown[1]. **c, d** DNA was isolated from 12 FFPE metastatic PCa samples and bisulfite converted together with control samples (human unmethylated and methylated DNA). Bisulfite converted promoter regions of *CDKN2A* and *STAT3* were amplified with methylation specific PCR primers. PMR (percentage of methylated reference) values were calculated as described in supplementary materials and methods by using methylated DNA as reference. Samples with a PMR lower than 10% were considered to be unmethylated. Each sample was run in duplicates. Mean values are shown; error bars: s.d. Data are shown in box and whisker plots with min to max values. Data were analyzed by One-way ANOVA with Tukey´s multiple comparison test.

a

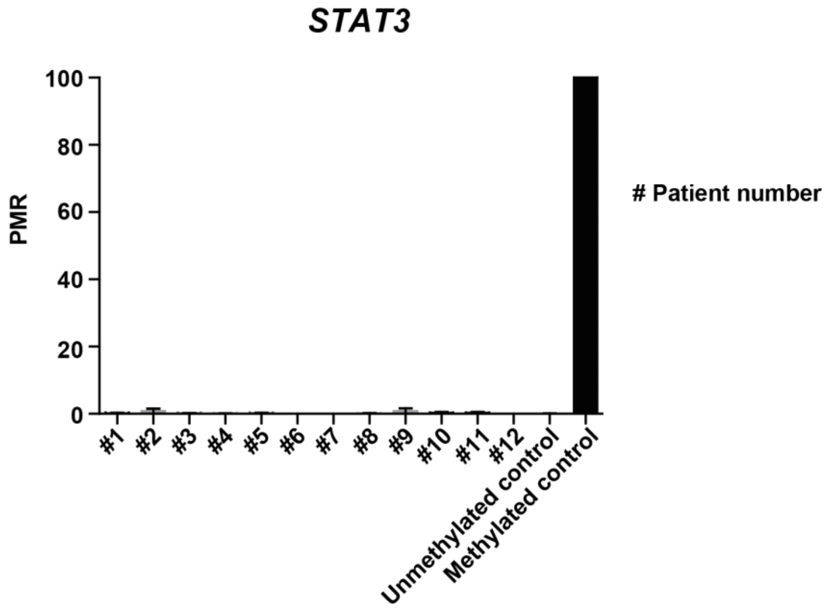

b

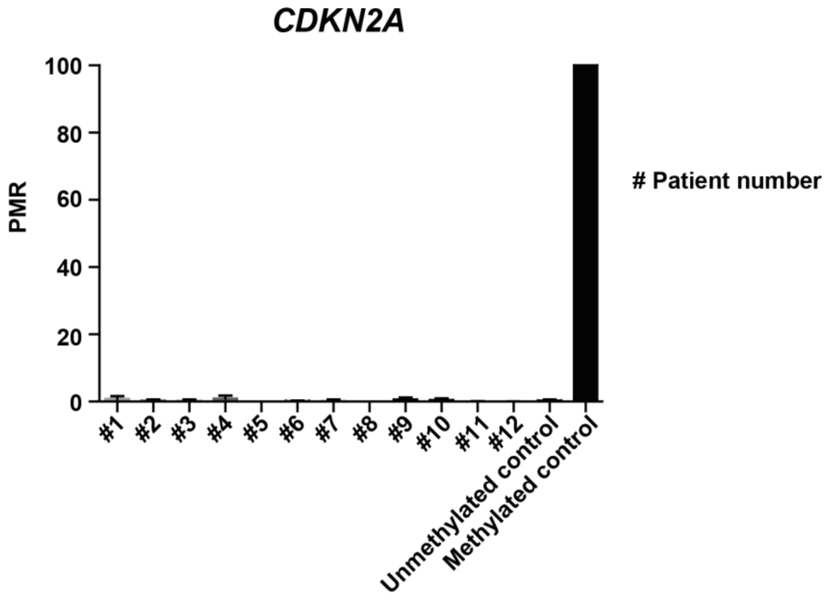

Supplementary Figure 14: Methylation of *STAT3* and *CDKN2A* promoter regions in primary PCa.

a, b Methylation of *STAT3* and *CDKN2A* promoter regions in primary PCa analyzed with the Illumina GoldenGate methylation array. *GSTP1* methylation is shown as an example for a hypermethylated marker in PCa. Normalized data from GEO methylation database were analyzed for each sample and were further normalized such that each intensity value was greater than zero (by adding the two smallest, positive intensities to each value) and log^{10}-transformed. Data were verified from the Gene Expression Omnibus (GEO) with accession number GSE38240 and GSE26126. Mean values are shown; error bars: s.d.

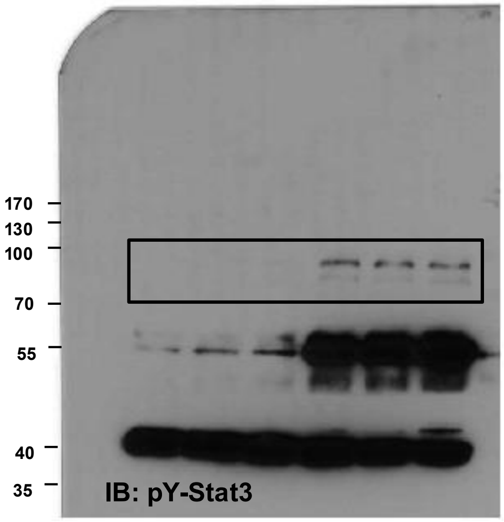

For Figure 1b

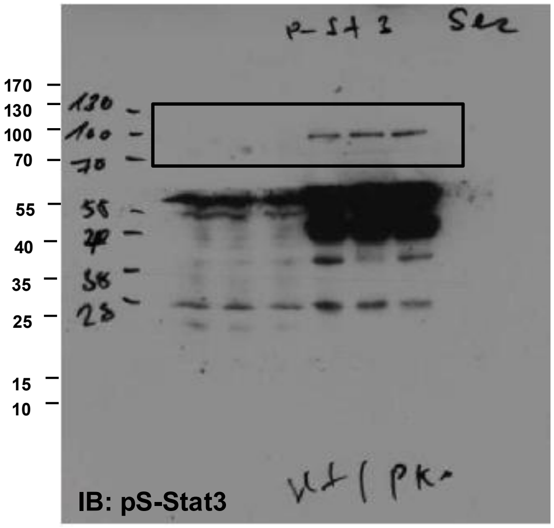

For Figure 1b

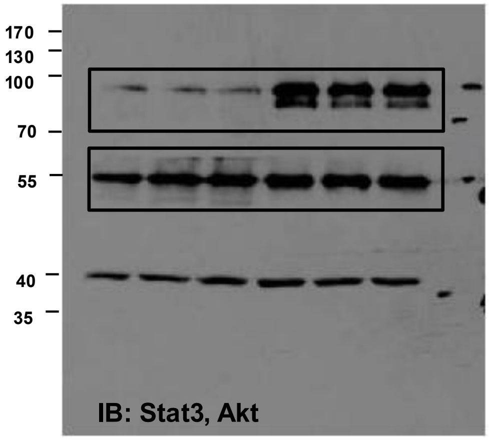

For Figure 1b

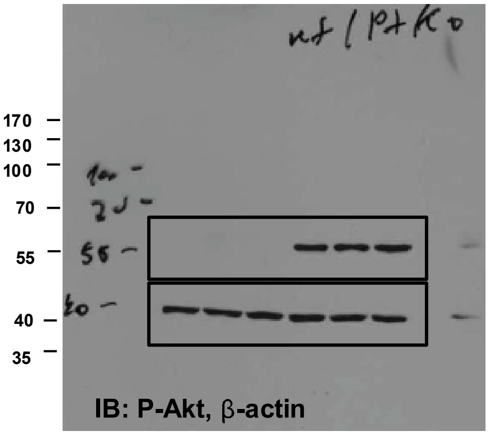

For Figure 1b

Supplementary Figure 15: Full scans of western blot data shown in Figure 1b

Original photos of immunoblots. Black boxes indicate the cropped areas displayed in the indicated figures.

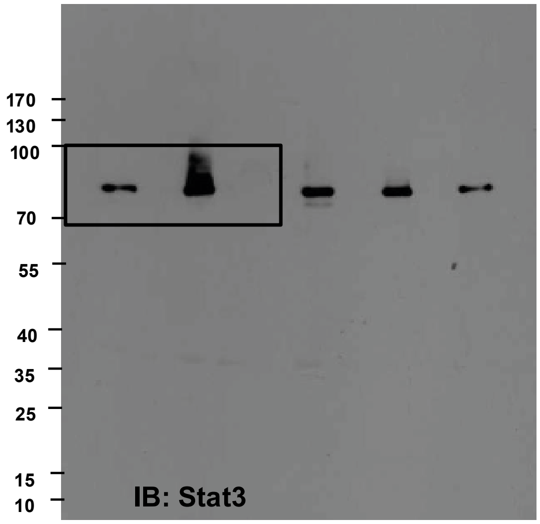

For Figure 3a

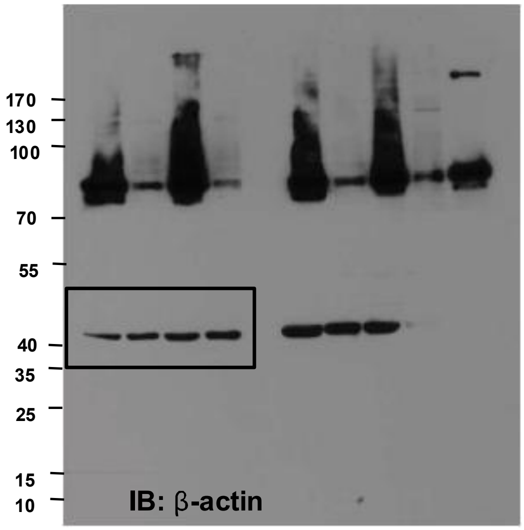

For Figure 3a

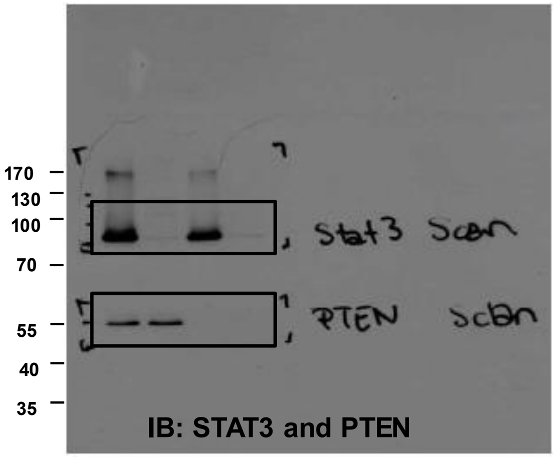

For Figure 3e

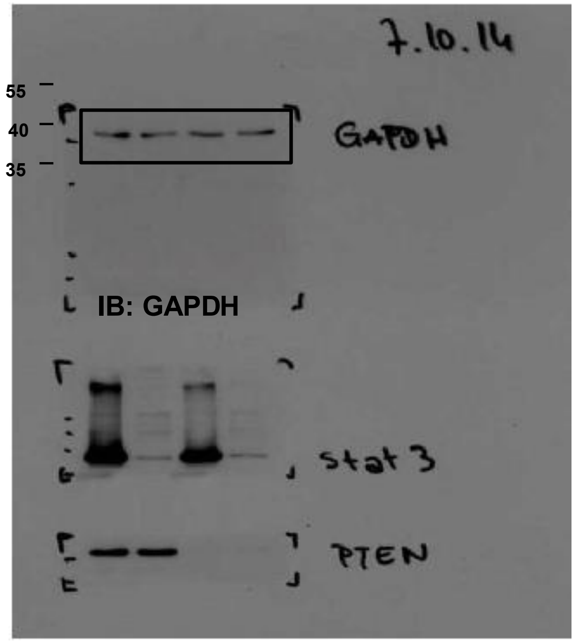

For Figure 3e

Supplementary Figure 16: Full scans of western blot data shown in Figure 3a, 3e
Original photos of immunoblots. Black boxes indicate the cropped areas displayed in the indicated figures.

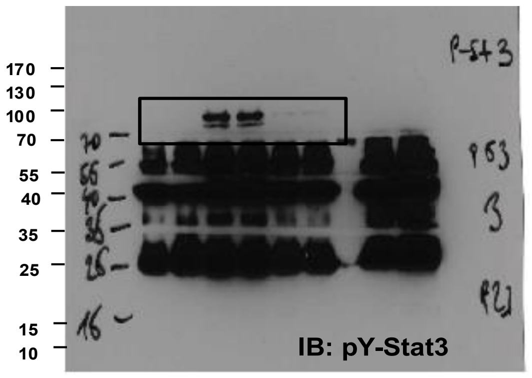

For Figure 4b

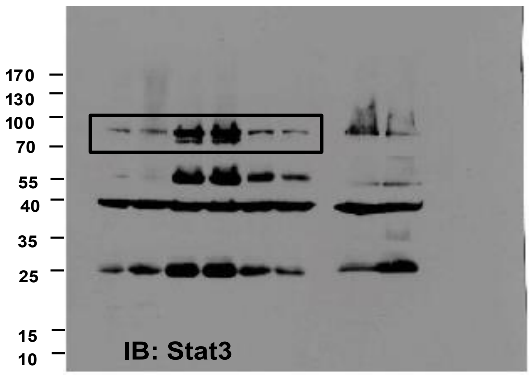

For Figure 4b

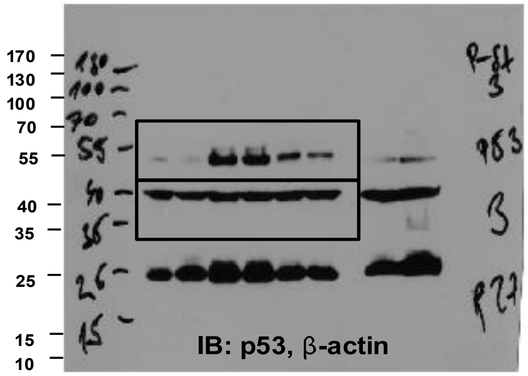

For Figure 4b

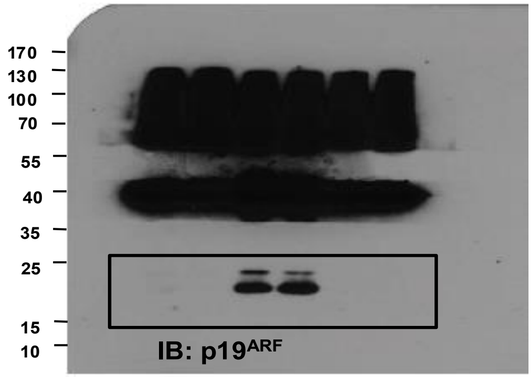

For Figure 4b

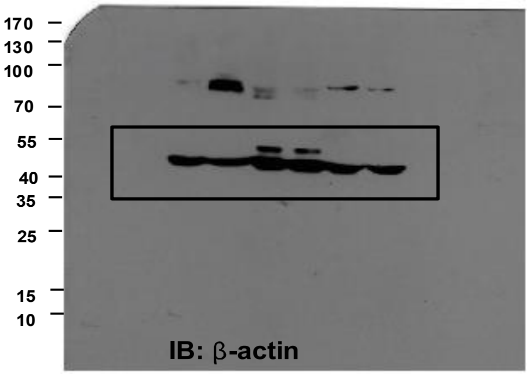

For Figure 4b

Supplementary Figure 17: Full scans of western blot data shown in Figure 4b

Original photos of immunoblots. Black boxes indicate the cropped areas displayed in the indicated figures.

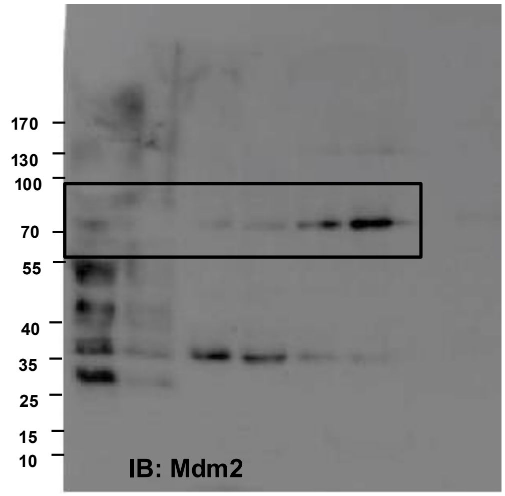

For Figure 4b

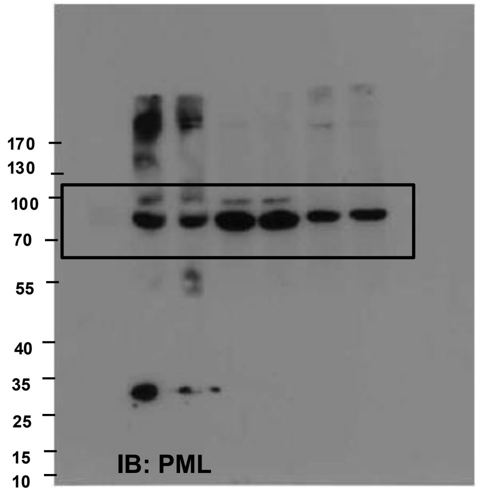

For Figure 4b

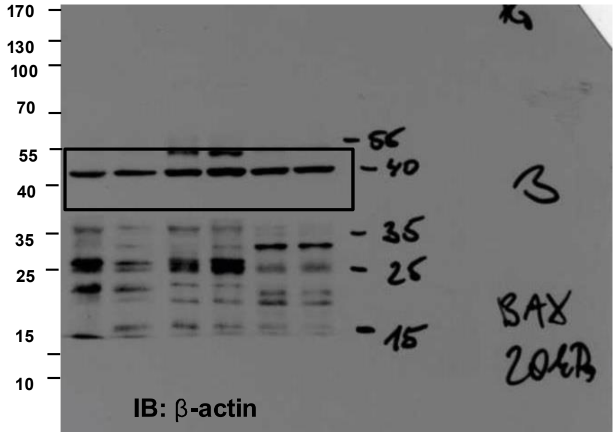

For Figure 4b

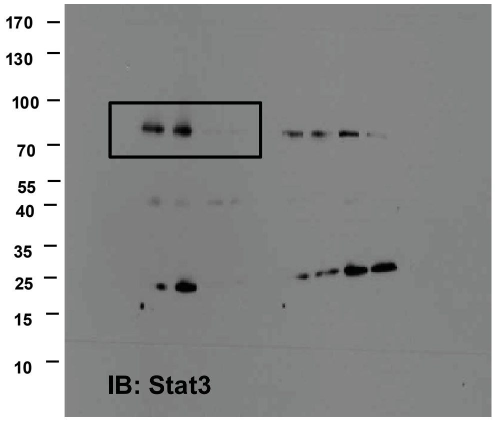

For Figure 4c

Supplementary Figure 18: Full scans of western blot data shown in Figure 4b, 4c
Original photos of immunoblots. Black boxes indicate the cropped areas displayed in the indicated figures.

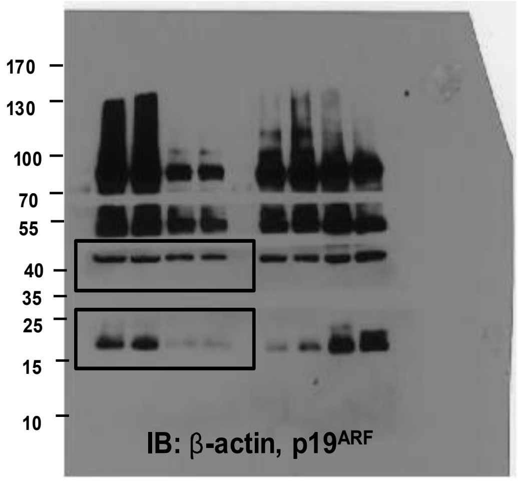

For Figure 4c

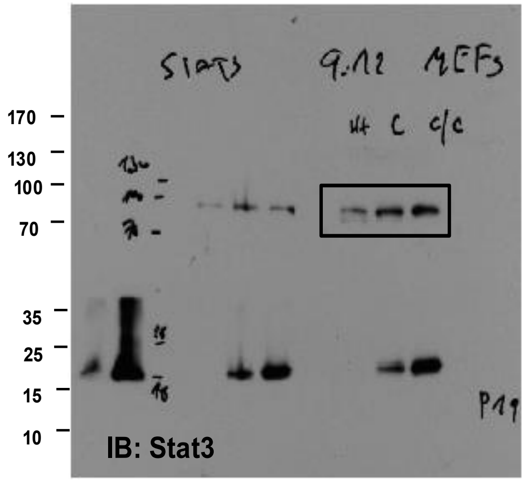

For Figure 4e

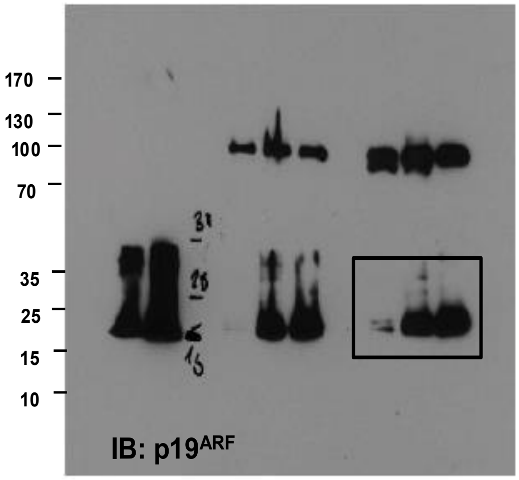

For Figure 4e

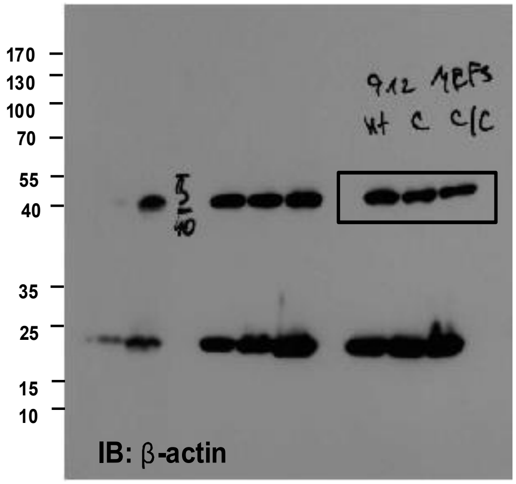

For Figure 4e

Supplementary Figure 19: Full scans of western blot data shown in Figure 4c, 4e
Original photos of immunoblots. Black boxes indicate the cropped areas displayed in the indicated figures.

Supplementary Table 1

The frequency of local and distant metastasis of each genotype to organs

WT	*Stat3*$^{pc-/-}$	*Pten*$^{pc-/-}$	*Pten*$^{pc-/-}$*Stat3*$^{pc-/-}$
0/13	0/8	1/12 to Seminal Vesicle	7/16 to Lung
			3/16 to Liver
			2/16 to Seminal Vesicle
			1/16 to Kidney
			1/16 to Oesophagus

Supplementary Table 2

PCR primer sets for detection of Stat3 binding sites in the p19^{ARF} and Socs3 promoter region

Primer	Sequence (5´-3´)
p19^{ARF1} forward	AATCCTGCACCGAGAAAGCA
p19^{ARF1} reverse	GCTACCCGATAGCAAGCACT
p19^{ARF2} forward	GGTTGAGGAGGACCGTGAAG
p19^{ARF2} reverse	CCACGAGGTAGAAGCAGGAC
Sosc3 forward	GGCCCCAGTCTGAGTATGAC
Socs3 reverse	ATTTCGCTTCGGGACTAGGT

Supplementary Table 3

qRT-PCR primer list

Primer	Sequence (5´-3´)
Socs3 forward	AGGCCGGAGATTTCGCTTCGG
Socs3 reverse	TTGCTGTGGGTGACCATGGCGC
Stat3 forward	AATGTCCTCTATCAGCACAACC
Stat3 reverse	TCTCCACCACCTTCATTTTCTG
β-actin forward	GCTCATAGCTCTTCTCCAGGG
β-actin reverse	CCTGAACCCTAAGGCCAACCG
IL-6Rα forward	ACACACTGGTTCTGAGGGAC
IL-6Rα reverse	TACCACAAGGTTGGCAGGTG
IL-6 forward	GAGGATACCACTCCCAACAGACC
IL-6 reverse	AAGTGCATCATCGTTGTTCATACA
$p19^{ARF}$ forward	CATGTTGTTGAGGCTAGAGAGG
$p19^{ARF}$ reverse	TGAGCAGAAGAGCTGCTACG
c-fos forward	CTGTCACCGTGGGGATAAAG
c-fos reverse	CCTACTACCATTCCCCAGCC

Supplementary Methods

Antibody validation for human TMA analysis

In order to evaluate $p14^{ARF}$ and STAT3 antibodies in immunohistochemistry under the same conditions as for TMA analysis human xenografted tumors were used. For STAT3 FFPE embedded SW620 colon cancer xenografts with shRNA mediated STAT3 knockdown were evaluated. STAT3-targeted and non-targeting controls were sectioned and stained. Efficient knockdown was verified by qRT-PCR (data not shown). The specificity of the $p14^{ARF}$ antibody was verified using human melanoma cell lines (VM-7, VM-28)[2] grown as xenografts and processed as described above. VM-7 harbors a deletion at the $p14^{ARF}$ locus, whereas VM-28 is wild type for $p14^{ARF}$.

siRNA transfections

Knockdown experiments were performed using siRNA duplexes (Dhamarcon) to silence PTEN and STAT3 by standard transfection protocols (RNAiMax, Qiagen). A non-targeting pool siRNA (Dharmacon) served as negative control. Effective knockdown was confirmed by Western blot.

Anchorage independent growth assays

For colony formation assays, 1×10^4 cells were seeded in 12-well plates in 0.4% select agar on top of 0.5% bottom select agar (Life Technologies) according to standard protocols of anchorage independent growth assays [3]. PCa cells were grown in 3D cultures at 37°C in a humidified atmosphere containing 5% CO_2 and clonogenic anchorage independent growth was monitored for 3 weeks. Colony formation was documented on a stereomicroscope with the Cell^D Image capture system (Olympus) and quantified using Colony Counter Software (MicrotecNition).

Lentiviral transduction and shRNA mediated knockdowns

Lentivirus transduction and subsequent production of cells for shRNA knockdown was carried out as described previously [4]. The following shRNA constructs were used: shRNA against Stat3 (shStat3) (TRCN0000071456) and control scrambled shRNA (shControl) (Sigma Aldrich mission TRC library).

Efficient knockdown of target transcripts was validated by qRT-PCR and Western blot analysis. Stably transduced knockdown and control cells were generated by selection in puromycin (1 mg/mL) prior to further analysis.

Invasion assays of *Pten$^{-/-}$* and *Pten$^{-/-}$shStat3* mouse prostate epithelial cell lines

Cells were stably transduced with lentiviral shRNAs targeting Stat3 or non-target control shRNAs and subjected to a matrigel invasion assay. After 48 hours of invasion, cells were fixed in methanol p.a. and stained with 1% Toluidine blue. Average invasive cell numbers are shown for 3 independent experiments. (each $p < 0.05$).

Organotypic invasion assay

Primary mouse prostate fibroblasts (8.5×10^4 cells/ml) were embedded into a collagen type I, rat tail (Corning B.V., Prod# 354236, final concentration 2 mg/ml) gel and allowed to contact the free floating gel for 7 days. Subsequently, tumor cells were seeded on top of the gel to form a confluent cell layer. Following overnight attachment, the gels were transferred to a stainless steel grid. An air/liquid interphase was created with epithelial growth medium to promote tumor cell invasion into the organotypic matrix. After 8 days, gels were fixed using Histofix (Lactan P087.5) and processed for H&E and IHC stainings.

Transfection

STAT3 negative PC3 cell line is derived from a PCa metastasis[5]. The PC3 cells were grown in DMEM containing 10% FCS and 1% Pen/Strep. 10^5 cells were plated in 24-well plates for 24 h. 24 h later cells were transfected with pcDNA3-TOPO-STAT3-V5 or empty vector (pcDNA3-TOPO, pc3.1, or pc3.1-EGFP; all these control vectors induced negligible cellular responses) using lipofectamine (Invitrogen) or turbofect (Qiagen). For cellular proliferation assays transfected cells were left for 48 h. The cells were then re-seeded in 6 cm dishes and treated with 0.5 mg/mL G418 for 4 days; to enrich transfected cells. Cells were then fixed with methanol p.a. for 20 min at RT. Then Giemsa (1.25%) was added for 1 h to stain the cells. These were then washed with H_2O and counted. For Western blot analysis cells were

transfected for 48 h. Cells were harvested and lysed in loading buffer (95°C; 100 µL/well; Roth) and sonicated twice.

In vivo **chromatin immunoprecipitation (ChIP) assays**

Single cell suspensions were prepared from 0.5 g of tumor tissue (isolated from *Pten*$^{pc-/-}$ or *Pten*$^{pc-/-}$*Stat3*$^{pc-/-}$ mice at 19 weeks of age). All buffers were kept at 37°C. Tumors were transferred to sterile Petri dishes, minced into little pieces using forceps and scissors. 4 ml of Accutase (PAA, L11-00) was added, vortexed and the samples were incubated for 5 min at 37°C until supernatants turned turbid. After brief incubation, supernatants were transferred into 50 ml Falcon tubes and 7 ml FBS-containing culture medium (10% of FBS) was added. This procedure was repeated two more times. Finally, cell suspensions were filtrated through a 40 µm cell strainer before fresh growth medium was added.

Single cell suspensions of mouse PCa tumor cells were crosslinked with 1% formaldehyde solution (1% formaldehyde in 50 mM Hepes pH 7.4, 100 mM NaCl, 1 mM EDTA, 0.5 mM EGTA) for 10 minutes, quenched with 0.125 M glycine for 5 minutes and harvested afterwards. Cell pellets were resuspended in LB1 buffer (50 mM Hepes pH 7.6, 140 mM NaCl, 1 mM EDTA, 10% glycerol, 0.5% NP-40, 0.25% Triton X-100) to lyse the cytoplasm. Nuclei were washed once in LB2 buffer (10 mM Tris-HCl pH 8.0, 200 mM NaCl, 1 mM EDTA, 0.5 mM EGTA), before lysis in LB3 buffer (10 mM Tris-HCl pH 8.0, 200 mM NaCl, 1 mM EDTA, 0.5 mM EGTA, 0.1% Na-Deoxycholate, 0.5% N-lauroylsarcosine). All buffers were complemented with 1 mM EDTA, 1 mM EGTA, 1 mM DTT, 50 mM NaF, 1 mM Na_3VO_4 and protease inhibitors (cOmplete, Mini, EDTA-free, Roche) before use. Fixed chromatin samples were fragmented using a COVARIS S2x sonicator (10 cycles in total; 1 cycle: 50 dc, 10 i, 500 cbp) at 4°C. Immediately after sonication, 0.5% Triton X-100 was added to the samples to aid solubilization of sheared DNA. Samples were cleared by centrifugation at 10,000 x g for 10 minutes. The supernatant was incubated overnight with 5 µg of Stat3-ChIP antibody (Cell Signaling, 12640) at 4°C on a rocking platform. Antibody-chromatin complexes were collected using Dynal protein G magnetic beads (NOVEX, Life technologies) and washed 5 times with wash buffer (50 mM Hepes-KOH pH 7.4, 500 mM LiCl, 1 mM EDTA, 1% NP-40, 0.7% Na-Deoxycholate). Bound material was released using Elution buffer (50 mM Tris-HCl pH 8.0, 10 mM EDTA, 1% SDS) at 65°C for 10 min. DNA-protein crosslinks were reversed by incubating the

samples overnight at 65°C. Samples were then treated with RNaseA and Proteinase K and purified using a QIAquick PCR cleanup column (Qiagen). Specific enrichment of isolated DNA was measured by qPCR using primers designed to amplify Stat3-binding sites in *p19*^{ARF} and *Socs3* promoter sequences (detailed information is given in **Supplementary Table 2**). Relative enrichment of chromatin precipitated with Stat3 antibody was normalized to the amount of DNA precipitated with IgG control antibody using the comparative C_T values. C_T values were determined by choosing threshold values in the exponential range of amplification curves.

Identification of putative Stat3 binding sites

Stat3 binding elements in the Mus musculus *p19*^{ARF} promoter were identified *in silico* by established methods[6]. Briefly, promoter regions of p19^{ARF} were scanned for potential binding motifs of the Stat3 using MAPPER2. We determined and confirmed by *in vivo* ChIP analysis that Stat3 is binding to putative binding sites at positions -787 and -210 upstream of the transcription initiation site (shown in **Supplementary Fig. 7e**).

Analysis of *IL-6* and *STAT3* mRNA expression in human tumor samples

To assess the prognostic significance of *IL-6* and *STAT3* expression in human tumor samples, the Taylor datasets[1,7] were interrogated. Z scores for *IL-6*/*STAT3* expression in all primary tumor samples were generated by comparison of *IL-6*/*STAT3* expression in normal prostate samples. Tumors with mRNA expression levels showing z-scores of less than -2 or greater than 2 were defined as being low in *STAT3* or high in *IL-6* expression, respectively. Graphpad Prism 6 software (GraphPad Software) was used to perform Kaplan-Meier analysis of survival of samples with altered *IL-6* and *STAT3* levels. We used time to BCR and censoring for the plots in **Fig. 7 a, b**. A log-rank test was used to report significance; the Hazard Ratio (HR) and its confidence interval were calculated using the Mantel-Haenszel method.

Xenograft models in NSG mice

LNCAP cells were grown in RPMI with 10% FBS, 1% Pen/Strep. Cells were split 24 h before harvesting: cells were detached with trypsin, washed twice in PBS and counted. Cells were suspended in PBS at 5 x 10^6/ml, mixed 1:1 with Matrigel (Corning), and kept on ice until injection. Nine weeks old NSG mice received sub-cutaneous injections of 200 µl tumor matrigel mix, so that each mouse received 5 x 10^5 cells. On day 2 mice received either oral gavage containing Ruxolitinib dissolved in a PBS/DMSO solution (20% DMSO), at 50 mg/kg; or a control PBS/DMSO (Ruxolitinib *n*=3; Control *n*=3). Mice were treated every subsequent day for a total of 10 days. Mice were monitored for tumor development and sacrificed 24 days into the experiment when one of the tumors had exceeded the size limit of 1.2 cm in diameter. Tumors were dissected and weighed, then fixed in formalin (10%) for further analysis.

Isolation of genomic DNA from FFPE tissue

DNA isolation from FFPE tissue (12 PCa metastases) was performed with the QIAamp DNA Blood Mini Kit according to protocol. FFPE tissue was macrodissected, deparaffinized with xylol, washed with ethanol, dried and resuspended in buffer ATL. Then, the suspension was incubated at 56°C with Proteinase K for 3 days and further processed according to protocol. DNA was eluted two times with 50 µl of sterile water.

Methylation specific PCR

0.5 µg of each DNA sample (12 PCa metastases), SssI treated control DNA (Zymo Research) and unmethylated control DNA (Zymo Research) were bisulfite-converted with the EZ DNA MethylationTM Kit (Zymo Research) according to the manufacturer´s protocol. Overnight conversion was carried out in a thermocycler at 95°C for 30 sec, 50°C for 60 min (16 cycles). Then, bisulfite DNA was eluted in 40 µl of sterile water and diluted to 200 µl and three consecutive dilutions (1:25) of SssI bisulfite DNA were prepared to obtain four methylated DNA standards. Methylation-specific primers were taken from[8] or designed with MethPrimer (http://www.urogene.org/methprimer). PCRs were performed according to the

MethyLight protocol[9] by using SYBR green instead of Taqman probes for quantification. Each PCR reaction consisted of 7.5 µl Sybr Fast mastermix (Peqlab), 2.5 µl primers (ALU or gene-specific, 100 µl stock primers were diluted 1:150), 2 µl of bisulfite converted DNA and 4.25 µl of sterile water. Standard curves were run with ALU primers and sample DNA was run with both ALU and gene-specific primers on a Biorad cycler in duplicates. PMR (percentage of methylated reference) values were calculated according to the following formula: [(SQSampleGene/SQSampleAlu)/(SQSssIGene/SQSssIAlu)]*100.

Supplementary References

1. Taylor, B.S., *et al.* Integrative genomic profiling of human prostate cancer. *Cancer Cell* **18**, 11-22 (2010).
2. Mathieu, V., *et al.* Aggressiveness of human melanoma xenograft models is promoted by aneuploidy-driven gene expression deregulation. *Oncotarget* **3**, 399-413 (2012).
3. Eberl, M., *et al.* Hedgehog-EGFR cooperation response genes determine the oncogenic phenotype of basal cell carcinoma and tumour-initiating pancreatic cancer cells. *EMBO Mol Med* **4**, 218-233 (2012).
4. Kasper, M., Regl, G., Eichberger, T., Frischauf, A.M. & Aberger, F. Efficient manipulation of Hedgehog/GLI signaling using retroviral expression systems. *Methods Mol Biol* **397**, 67-78 (2007).
5. Clark, J., *et al.* Genome-wide screening for complete genetic loss in prostate cancer by comparative hybridization onto cDNA microarrays. *Oncogene* **22**, 1247-1252 (2003).
6. Riva, A. The MAPPER2 Database: a multi-genome catalog of putative transcription factor binding sites. *Nucleic Acids Res* **40**, D155-161 (2012).
7. Wilt, T.J., *et al.* Systematic review: comparative effectiveness and harms of treatments for clinically localized prostate cancer. *Ann Intern Med* **148**, 435-448 (2008).
8. Weisenberger, D.J., *et al.* Analysis of repetitive element DNA methylation by MethyLight. *Nucleic Acids Res* **33**, 6823-6836 (2005).
9. Campan, M., Weisenberger, D.J., Trinh, B. & Laird, P.W. MethyLight. *Methods Mol Biol* **507**, 325-337 (2009).
